# PSMD11 modulates circadian clock function through PER and CRY nuclear translocation

**DOI:** 10.1371/journal.pone.0283463

**Published:** 2023-03-24

**Authors:** Sibel Cal-Kayitmazbatir, Lauren J. Francey, Yool Lee, Andrew C. Liu, John B. Hogenesch

**Affiliations:** 1 Divisions of Human Genetics and Immunobiology, Department of Pediatrics, Cincinnati Children’s Hospital Medical Center, Cincinnati, Ohio, United States of America; 2 Department of Systems Pharmacology and Translational Therapeutics, Institute for Translational Medicine and Therapeutics, Perelman School of Medicine, University of Pennsylvania, Philadelphia, Pennsylvania, United States of America; 3 Department of Translational Medicine and Physiology, Elson S. Floyd College of Medicine, Washington State University, Spokane, Washington, United States of America; 4 Department of Physiology and Aging, University of Florida College of Medicine, Gainesville, Florida, United States of America; Karlsruhe Institute of Technology, GERMANY

## Abstract

The molecular circadian clock is regulated by a transcriptional translational feedback loop. However, the post-translational control mechanisms are less understood. The NRON complex is a large ribonucleoprotein complex, consisting of a lncRNA and several proteins. Components of the complex play a distinct role in regulating protein phosphorylation, synthesis, stability, and translocation in cellular processes. This includes the NFAT and the circadian clock pathway. PSMD11 is a component of the NRON complex and a lid component of the 26S proteasome. Among the PSMD family members, PSMD11 has a more specific role in circadian clock function. Here, we used cell and biochemical approaches and characterized the role of PSMD11 in regulating the stability and nuclear translocation of circadian clock proteins. We used size exclusion chromatography to enrich the NRON complex in the cytosolic and nuclear fractions. More specifically, *PSMD11* knockdown affected the abundance of PER2 and CRY2 proteins and the nuclear translocation of CRY1. This changed the relative abundance of CRY1 and CRY2 in the nucleus. Thus, this work defines the role of PSMD11 in the NRON complex regulating the nuclear translocation of circadian repressors, thereby enabling cellular circadian oscillations.

## Introduction

Circadian clocks are internal timing systems that can adapt to environmental changes in regulating 24 h behavior and physiology. Many organisms from single-cell bacteria to humans have biological clocks and exhibit physiological rhythms [1]. In eukaryotes, the transcriptional translational feedback loop (TTFL) regulates the internal molecular clock [2]. In mammals, BMAL1 and CLOCK are positive regulators that form a heterodimer and initiate the expression of clock-controlled genes, including their own transcriptional repressors (*CRY1*, *CRY2*, *PER1*, and *PER2*) [3]. PER and CRY proteins translocate to the nucleus to repress BMAL1-CLOCK transcriptional activity [4]. Although the genetic mechanism of the TTFL is widely accepted, the biochemical mechanisms that fine-tune clock are complex and not well defined.

Several post-translational mechanisms are known to regulate circadian timekeeping, including acetylation [5] and SUMOylation [6] of BMAL1; ubiquitination of CRY1, CRY2, PER1, PER2, REV-ERBɑ and BMAL1 [7–10], and phosphorylation of both negative and positive regulators [11]. In recent years, the majority of rhythmic phosphorylation events are detected on non-rhythmic proteins [12]. This means post-translational modifications (PTMs) by themselves can start cellular rhythmic events. In addition to PTMs, interactions with other proteins can also change the activity and/or cellular localization of the clock proteins. For example, cystathionine-*β*-synthase (CBS) interacts with CRY1 and changes its repression activity [13]. KPNB1 interacts with PER proteins and regulates the translocation of the PER/CRY complex into the nucleus [14]. While dietary changes affect CBS expression/activity and oncogenicity upregulates KPNB1 expression, protein-protein interactions are also important for maintaining homeostasis under different environmental conditions [15, 16].

The NRON complex is a large ribonucleoprotein complex including several proteins with distinct roles. In 2005, we found a long non-coding RNA that represses NFAT (nuclear factor of activated T cells) and named it NRON (non-coding repressor of NFAT). NRON interacts with multiple proteins with distinct roles and was speculated to regulate NFAT nuclear trafficking [17]. Subsequent experiments using size exclusion chromatography and flow cytometry demonstrated that nuclear translocation of NFAT is in fact regulated by the NRON complex [18]. This showed that NRON acts as a scaffold together with IQGAP1 and calmodulin, for the kinases CSNK1, GSK3*β*, and DYRK1A to phosphorylate NFAT. Importantly, many members of the NRON complex are known to interact with core clock proteins and play important roles in the nuclear translocation of the repressor complex [14, 19].

Here, we show that the NRON complex regulates the nuclear translocation of key proteins in the circadian clock. First, we analyzed the role of PSMDs in regulating cellular circadian clocks. We found that knockdown of *PSMD11* ablated cellular rhythms. While *PSMD4* and *PSMD12* knockdown did not affect rhythmicity, they altered the baseline output. Further, we show that PSMD11 controls PER2 and CRY2 proteasomal degradation. We enriched the NRON complex with size exclusion chromatography (SEC) and detected the complex components by Western blot analysis. More specifically, PSMD11, CRY1, and CRY2 were detected in the NRON complex together with IQGAP1, KPNB1, CSE1L, GSK3*β*, CSNK1*ε*, and CSNK1*δ*. We found that cytoplasmic and nuclear accumulation of the NRON complex is temporally controlled and PSMD11 regulates the nuclear translocation of the NRON complex components, including CRY1. The regulation is through direct interactions of PSMD11 with KPNB1 and CRY1, but not PER2 or CRY2. Collectively, these results highlight the importance of PSMD11 in regulating the circadian system. And, more broadly, the NRON complex regulates the stability and nuclear accumulation of circadian clock negative regulators and links the clock to other pathways.

## Results

### PSMD11, but not PSMD4 and PSMD12, regulates the cellular circadian clock

**Fig 1:**
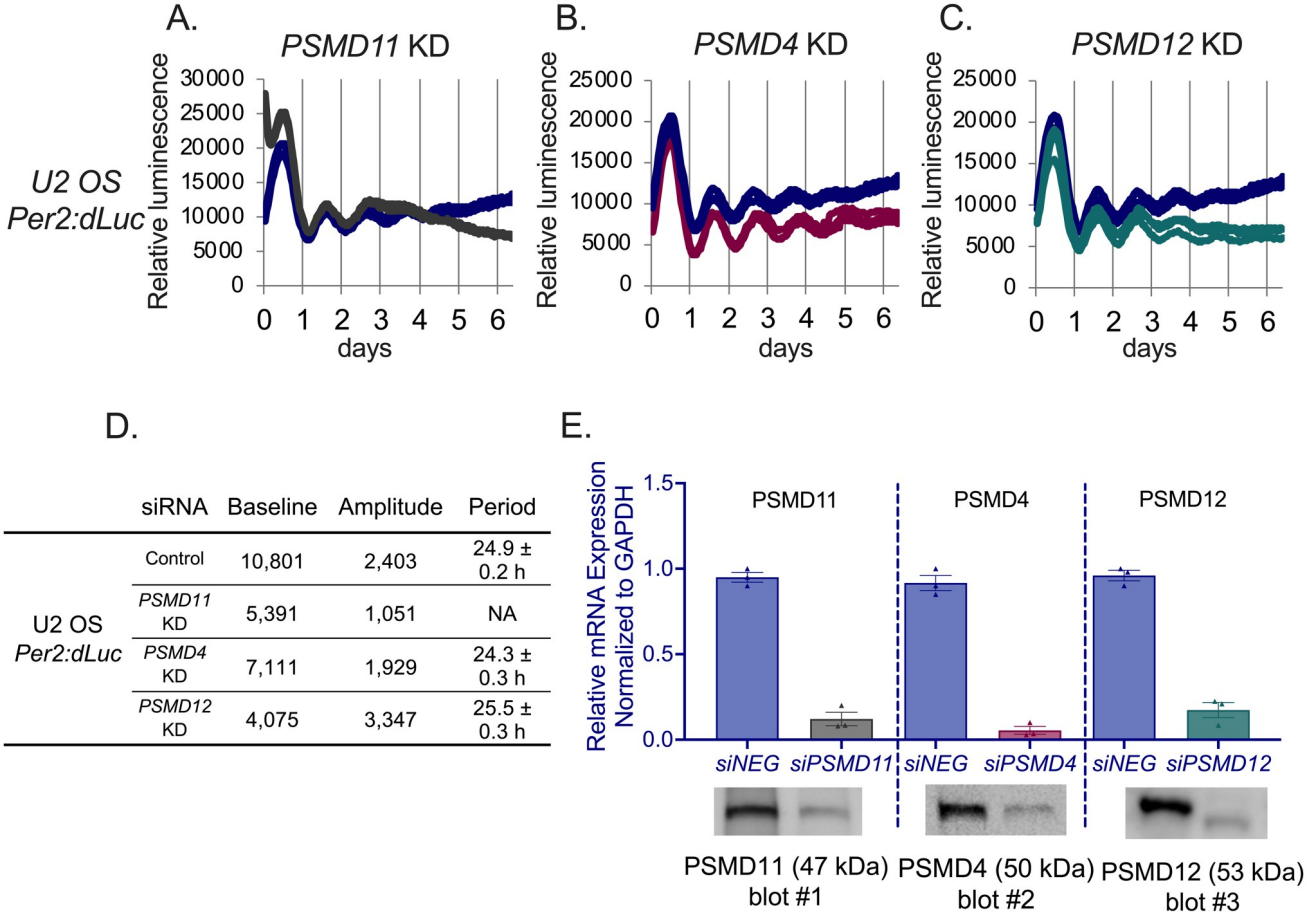
PSMD11 regulates cellular circadian clocks, but PSMD4 and PSMD12 do not. (A), (B), (C) PSMD11, PSMD4 and PSMD12 knockdown cellular circadian profiles. Representative bioluminescence records of circadian rhythms in U2 OS *Per2*:*dLuc* reporter cells. n = 3 independent experiments (D) Baseline, amplitude and phase results of A-C that are calculated with WaveClock algorithm. (E) mRNA levels detected with QPCR to check the RNAi knockdown effects. mRNA levels are normalized to GAPDH. Statistical analyses were performed with unpaired t tests. Error bars represent SEM. Representative Western blot images to confirm the protein level effect is shown below for each gene. Stain-free loading controls are shown in S1B Fig. Uncropped controls and images for each Western blot image are represented in ‘S1 Raw images’.

PSMD11 is a key component of the NRON complex and its knockdown affects the circadian clock of U2 OS cells [19, 20]. We aimed to determine the specific role of PSMD11 in regulating cellular circadian clocks. Among the many PSMD members, we selected *PSMD4* and *PSMD12* as comparators as they both are 26S proteasome lid components with functional domains to stabilize and activate the 26S proteasome. We examined the effects of *PSMD4*, *PSMD11*, and *PSMD12* knockdown on circadian rhythms in U2 OS *Per2*:*dLuc* reporter cells. While knockdown of *PSMD11* caused arrhythmia after the first 1.5 days (period couldn’t be calculated for *PSMD11* KD), *PSMD4* and *PSMD12* did not disrupt rhythmicity like PSMD11 (Fig 1A). We should note that the first day is when the cells are adjusting to the new media with the synchronizing agent (dexamethasone) [21]. Baseline, amplitude and period of the luminescence data was determined through the WaveClock algorithm [22] (Fig 1D). We detected the knockdown efficiencies with QPCR and verified them with Western blot (Fig 1E). These phenotypes were not due to cell death as measured with ATPlite luminescence assay. (S1A Fig). We also detected the circadian effects of the loss of *PSMD11*, *PSMD4*, and *PSMD12* on U2 OS *Bmal1*:*dLuc* reporter cells and detected that loss of *PSMD11* drastically dampened amplitude (S1C–S1G Fig). In conclusion, absence of *PSMD11* disrupts the circadian rhythms of the cells. Thus, we focused on the distinct role of *PSMD11* in regulating the circadian clock.

### PSMD11 regulates CRY2 and PER2 protein levels

**Fig 2:**
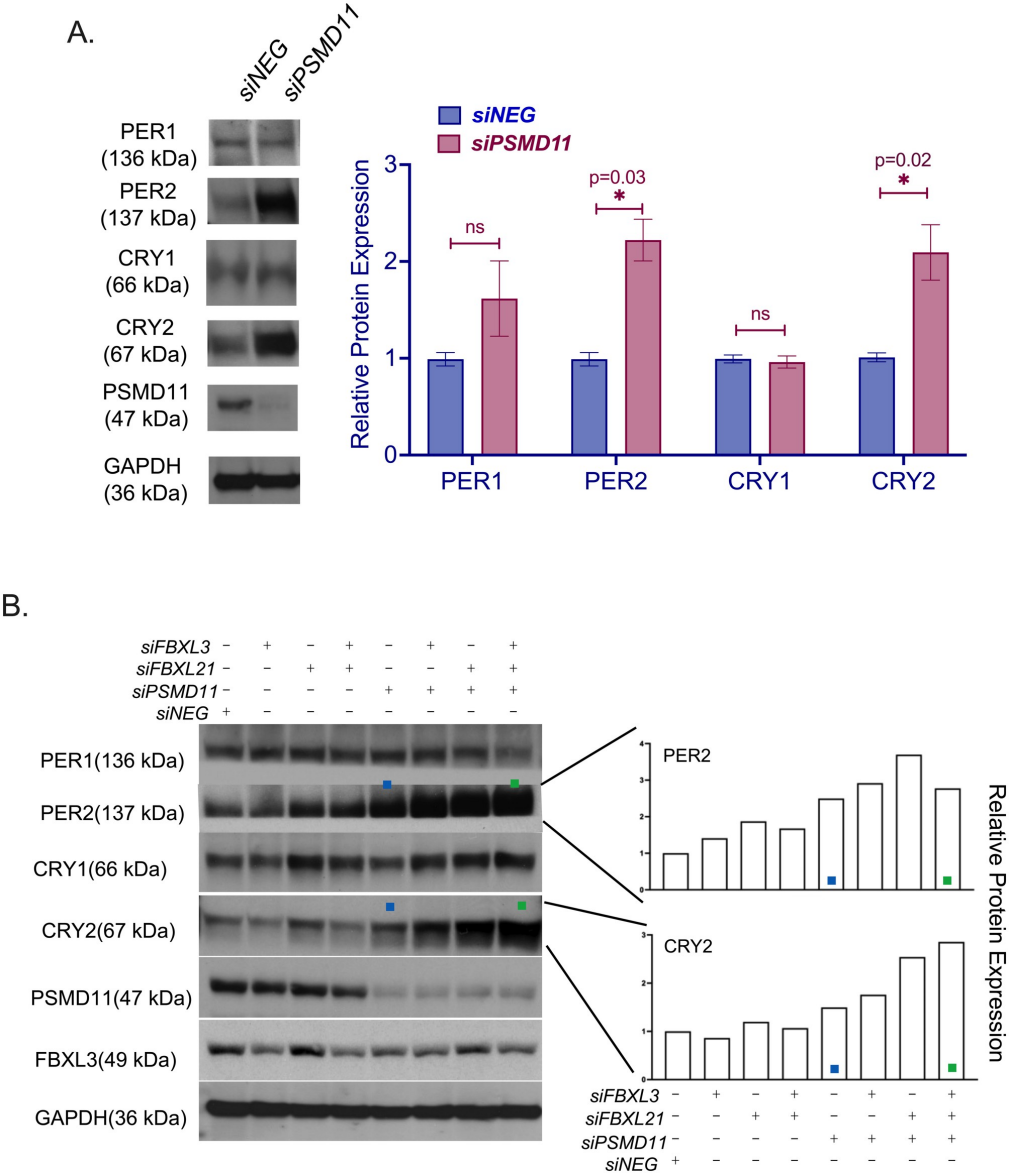
Proteasomal role of PSMD11 on circadian clock. (A) Absence of *PSMD11* increases PER2 and CRY2 protein levels. PER1, PER2, CRY1 and CRY2 levels are detected with Western blot when *PSMD11* is knocked down with an siRNA cocktail and control (*siNEG*) samples. n = 3, significancy is analyzed with unpaired t tests. (B) PSMD11 mediates regulation of PER2 and CRY2 abundances possibly through FBXL3/FBXL21. PER1, PER2, CRY1, and CRY2 levels are detected with Western blot when *FBXL3*, *FBXL21*, and/or *PSMD11* is knocked down with an siRNA cocktail. PER2 and CRY2 levels in *siPSMD11* (lane 5) are marked with blue squares and their levels when both FBXLs are knocked down along with *PSMD11* are marked with green squares (lane 8) for easier comparison. *FBXL21* mRNA levels are represented on S2C Fig. Quantification of CRY2 and PER2 on the represented blot is shown on the right. This experiment has one biological replicate. Experiments on this figure are performed in U2 OS cells.

The NRON complex has more than a dozen components, including nucleocytoplasmic transporters and kinases, as well as proteins responsible for protein synthesis and degradation. Knocking down the NRON complex components affected the cellular circadian clock [19]. Here, we show that knockdown of *NRON* led to a significant increase in CRY2 levels, but didn’t drastically affect PER1, PER2, or CRY1 (S2A Fig). We determined that a stable NRON complex and PSMD11 are required for CRY2 stability. In addition to CRY2, *PSMD11* knockdown also led to increased levels of PER2 (Fig 2A). We measured *CRY1*, *CRY2* and *PER2* mRNA levels by qPCR and *PSMD11* knockdown did not affect their mRNA levels (S2B Fig). This suggests that the effect of PSMD11 on the circadian clock is at the post-translational level. FBXL3 and FBXL21 are known to regulate CRY1 and CRY2 degradation [23]. Hence, we asked whether PSMD11 coordinates with FBXL3 and FBXL21 to regulate CRY1 and CRY2. We knocked down *FBXL3*, *FBXL21*, and *PSMD11* individually or in combination and assayed PER and CRY protein levels. While knockdown of *PSMD11* alone (marked with blue squares) increased PER2 and CRY2 levels, the combined knockdown of *PSMD11* with *FBXL3* and/or *FBXL21* (marked with green squares) led to a further increase in their levels (Fig 2B). We note the reduced FBXL3 level in *PSMD11* KD samples, that might be suggesting a genetic or a biochemical interaction between them. However, our data revealed the correlation between the reduced PSMD11 and increased CRY2 and PER2 levels (lane 2 vs lane 5). *FBXL21* knockdown led to increased CRY1 levels, but *PSMD11* knockdown did not have an additional effect. Quantification of the represented blot is shown on the right. In conclusion, proteasomal degradation of CRY2 and PER2 through PSMD11, but not CRY1, might be mediated by FBXL3 and FBXL21.

### Spatiotemporal regulation of the NRON complex in U2 OS cells

To study the spatiotemporal dynamics of the NRON complex, we used Jurkat and U2 OS cell models. We utilized Jurkat cells to optimize the enrichment of the NRON complex with size exclusion chromatography (SEC) [18]. We isolated native whole cell proteins from the cells and enriched the NRON complex with SEC (S3A Fig). We are aware that size exclusion chromatography has limitations in terms of determining the certain size [24]. Therefore, we mention the size of the complex as an approximate value detected relative to the gel filtration calibration kit (S3B Fig). We analyzed the fractions from the SEC column with Western blot and showed that IQGAP1, CRY1, CSNK1*ε*, and GSK3*β* migrated together in fractions 22–25 (S3C Fig). IQGAP1, CSNK1*ε*, and GSK3*β* have previously shown to be present at the same fraction and represent the NRON complex [18]. We concluded that these fractions represent the NRON complex that has a molecular weight of around 670 kDa and contains NRON complex components.

**Fig 3:**
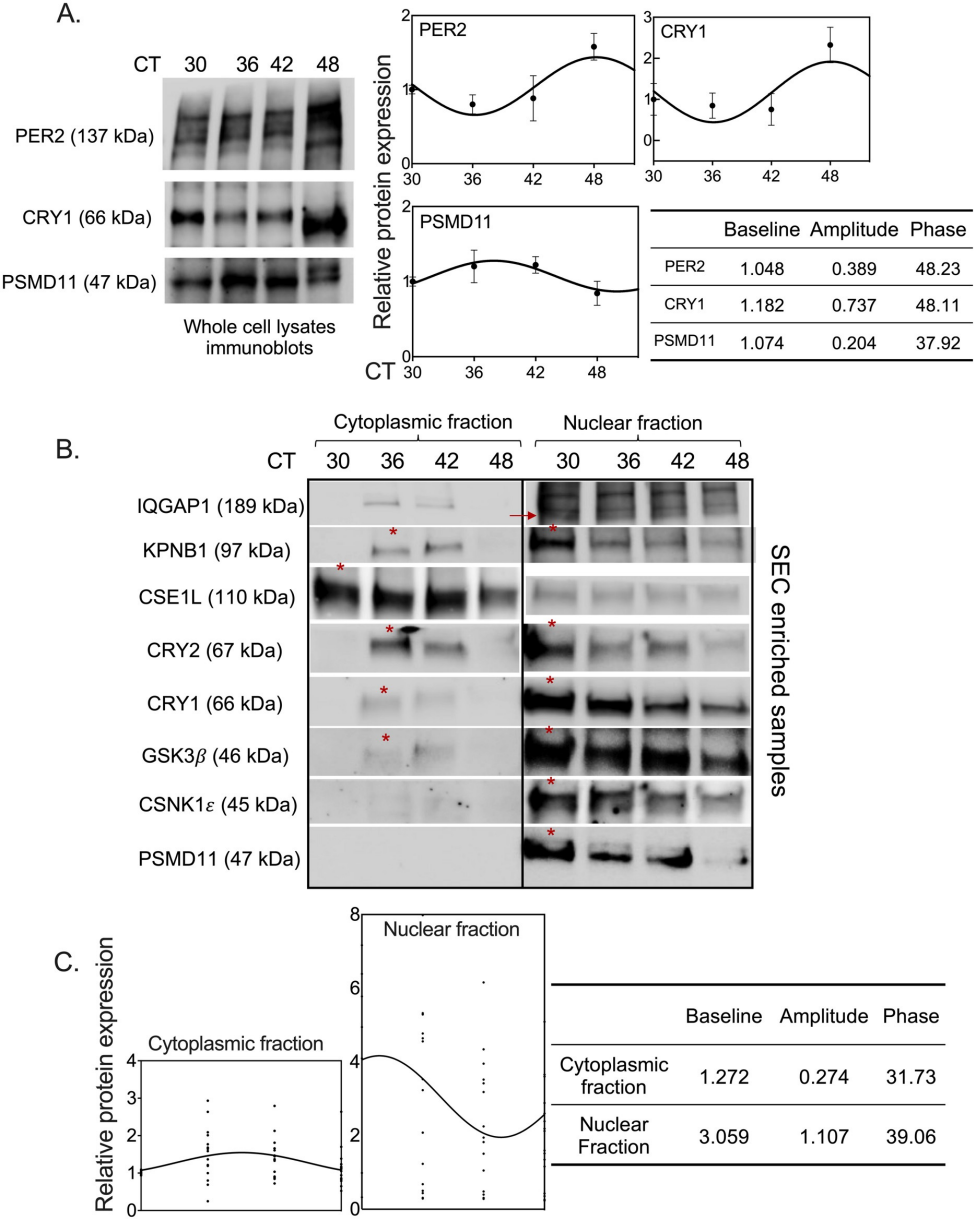
Temporal regulation of NRON complex at cytoplasmic and nuclear fractions. (A) PER2, CRY1 and PSMD11 protein levels in whole cell lysates from CT30, CT36, CT42 and CT48. Multiple bands in PER2 blot are caused by hyperphosphorylated PER2 proteins. Stain-free loading control is represented at S3D Fig. Quantifications of three independent experiments are represented on the right. Baseline, amplitude and phase are calculated with cosinor method. P-values for PER2, CRY1 and PSMD11 respectively; 0.06, 0.08, and 0.11 (B) Spatiotemporal analysis of NRON complex components in U2 OS cells. NRON complex component proteins -IQGAP1, KPNB1, CSE1L, CRY2, CRY1, GSK3β, CSNK1ε, and PSMD11- were detected in the fractions 22 to 25 in cytoplasmic and nuclear enriched samples at CT30, CT36, CT42 and CT48 by Western blot. Arrow indicates a non-specific band. Cytoplasmic and nuclear fractions for each protein are run on the same gel. Peak time point of the complex components at each cellular fraction is marked with red asterisks. (C) Quantification of all proteins detected relative to CT30 cytoplasmic fraction from two independent experiments is shown. Baseline, amplitude and phase are calculated with cosinor method. P-values for cytoplasmic fraction and nuclear fraction respectively; 0.019 and 0.0191.

Next, we moved to U2 OS cells to study the spatiotemporal dynamics of the NRON complex in the circadian clock mechanism. We detected rhythmic PER2, CRY1, and PSMD11 protein levels in whole cell lysates. Multiple bands in PER2 western blot demonstrate hyperphosphorylated forms of the protein as stated before in the literature [25]. Although it is not optimal to determine the precise phase, PER2 and CRY1 seemed to peak after PSMD11 (Fig 3A, see S3D Fig for loading controls). Quantification and cosinor analysis of the three independent experiments are shown on the right. We extracted native cytosolic and nuclear proteins from the cell pellets and examined the separation efficiency by Western blot (S3E Fig). Then, we focused on the proteins specifically in the NRON complex. For this, we applied SEC to the cytoplasmic and nuclear fractions at different circadian times (CT). We detected IQGAP1, KPNB1, CSE1L, CRY2, CRY1, GSK3*β*, CSNK1*ε*, and PSMD11 in the same fractions. We concluded that these proteins are associated with each other in the NRON complex (Fig 3B). Even though the total protein levels of CRY1 and PSMD11 were not in the same phase (Fig 3A), their levels in the NRON complex within the nuclear fraction peaked at CT30 (Fig 3B) reflecting their time and subcellular location specificity. These results are consistent with previous studies that detected direct interactions between IQGAP1, GSK3*β*, and CSNK1*ε* in the NRON complex [17, 18]. Strikingly, we detected CRY1, CRY2, GSK3*β*, CSNK1*ε*, and PSMD11 primarily in the nucleus (SEC enriched samples, Fig 3B). In Fig 3C, graphs represent the protein levels of all proteins detected in the NRON complex, relative to their levels in CT30-cytoplasmic fraction. Baseline, amplitude and phase are calculated with cosinor method. Overall, the baseline and amplitude of the proteins included in the NRON complex are enriched more in the nuclear fraction and display a higher amplitude than cytoplasmic fraction. GSK and CSNK1 proteins are known to be in the cytoplasm [26]. Our data showing that these proteins in the NRON complex are mainly localized in the nucleus further supports the role of the complex in nucleocytoplasmic trafficking of clock proteins. In both SEC inputs (S3F Fig) and SEC-enriched samples, CRY1 is higher in the nuclear than in the cytoplasmic fractions, which is consistent with a recent real-time fluorescence imaging study [27]. Overall, the complex in the cytoplasmic fraction peaked at CT36, whereas those in the nuclear fraction peaked at CT30. Thus, nucleocytoplasmic shuttling of the complex is temporally regulated in a circadian manner.

### PSMD11 regulates nuclear transport of NRON complex components, including CRY1

**Fig 4:**
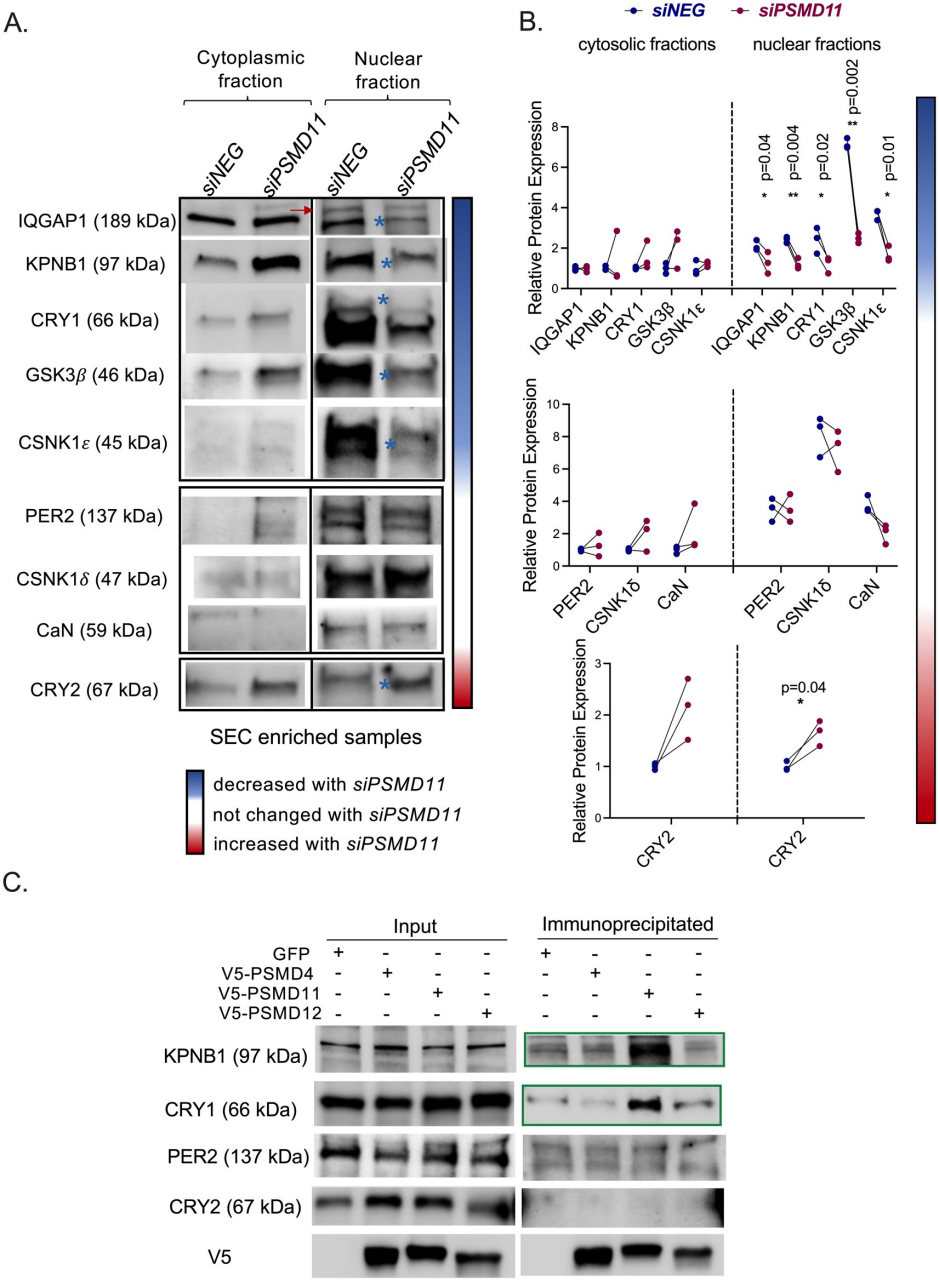
PSMD11 regulates circadian clock by regulating nuclear transport of the NRON complex. (A) Indicated proteins are detected in control (*siNEG*) and *siPSMD11* samples in SEC enriched cytoplasmic and nuclear fractions of unsynchronized U2 OS cells. Cytoplasmic and nuclear fractions for each protein are run on the same gel, then cut and pasted together for representation. Arrow indicates a non-specific band. Significantly affected blots by siPSMD11 are marked with blue asterisks on Western blot images. (B) Graphs represent Western blot quantifications of three independent experiments shown in (A). Significancy is analyzed with paired t test. P-values of the significant analyses are displayed on the graph. (C) Pulldown assay using whole cell lysates of U2 OS cells overexpressing PSMD-V5 plasmids or GFP. Pull down experiment repeated two times.

Next, we examined the effect of *PSMD11* perturbation on NRON complex formation and nucleocytoplasmic translocation. For this, we knocked down *PSMD11* and isolated the nuclear and cytoplasmic proteins, followed by enrichment of the NRON complex by SEC in unsynchronized U2 OS cells. Western blot analysis detected IQGAP1, KPNB1, CRY1, CRY2, GSK3*β*, CSNK1*δ*, CSNK1*ε*, PER2, and CaN (PPP3CA) in control (*siNEG*) and knockdown (*siPSMD11*) samples (Fig 4A). Consistently with the literature, we detected hyperphosphorylated PER2 proteins in the nuclear fraction, but not in the cytoplasmic fraction [25]. *PSMD11* knockdown significantly decreased the nuclear levels of IQGAP1, KPNB1, CRY1, GSK3*β*, and CSNK1*ε*. In contrast, PER2, CSNK1*δ*, and CaN levels were not affected, and CRY2 levels were higher (Fig 4A-bottom). Fig 4B represents quantification of the Western blot data. Significantly affected proteins in Fig 4A are marked with blue asterisks for clarification. Absence of *PSMD11* increased the abundance of CRY2 and PER2 in whole cell lysates (Fig 2B). However, in the nucleus, within the complex, *PSMD11* perturbation resulted in decreased levels of CRY1 and increased levels of CRY2. The ratio of nuclear CRY1:CRY2 is critical, as CRY1 is a much more potent CLOCK/BMAL1 repressor than CRY2 [28]. Since PSMD11 specifically regulates the nuclear translocation of CRY1, it likely functions to regulate the relative CRY1:CRY2 amounts in the nucleus and therefore the relative transcriptional activation and repression activities. The effect of the CRY1:CRY2 imbalance also helps to explain the disrupted phenotype in *PSMD11* knockdown cells (Fig 1A).

Post-translational modifications such as phosphorylation are known to regulate the stability and nuclear transport of core clock regulators like BMAL1 and PER2 [29, 30]. Although we observed an overall PER2 increase in cells, PER2 levels within the complex were not changed in *PSMD11* deficient cells. This may be caused at least in part by the reduced levels of the relevant kinases, particularly GSK3*β* and CSNK1*ε* in the nuclear complex (Fig 4A). Using co-IP and Western blot analyses, we showed that PSMD11 specifically interacted with KPNB1 and CRY1, not PER2 or CRY2 (Fig 4C-green boxes). Thus, PSMD11 regulates the nuclear transport of the NRON complex by interacting with KPNB1 and CRY1.

## Discussion

Properly timed nuclear localization of negative regulators PER and CRY is required for circadian clock function. The role of post-translational modifications (PTMs) on the molecular clockwork is appreciated, but not fully understood [31]. In addition to the PTMs, clock proteins are interpreted to be a part of a large protein complex to mutually regulate each other’s activity [32]. In a study published in 2017, researchers identified that circadian clock proteins are integrated in large flexible globular structures around 900 kDa [33]. So possibly, there are several protein complexes regulating intracellular trafficking and transcription for circadian clock. In another important study, investigators identified Mybbp1a protein as a CRY1 complex component. However, they did not detect PSMD11(47 kDa) because they analyzed proteins from 80 kDa to 200 kDa [34]. Here, we show that the NRON complex regulates the stability of CRY2 and PER2, and nuclear translocation of CRY1 through PSMD11.

To investigate the specific role of PSMD11, we included PSMD4 and PSMD12, other lid components of the 26S proteasome, as comparators. Of these, only *PSMD11* knockdown caused arrhythmic clock function (Fig 1). In the absence of *PSMD11*, less CRY1 is detected in the nuclear complex (Fig 3B) and more CRY2 accumulates there. In other words, knockdown of *PSMD11* changed the ratio of the components of the repressor complex in the nucleus and consequently, disrupted circadian clock function (Fig 1A).

KPNB1 enables NFAT nuclear entry and is required for PER/CRY nuclear import [14, 18]. We showed that KPNB1 also interacts with PSMD11. We conclude that both KPNB1 and PSMD11 are prominent in regulating nuclear translocation of circadian clock negative regulators as part of the NRON complex. While KPNB1 regulates the nuclear transport of the PER/CRY heterodimer, PSMD11 regulates the nuclear transport of CRY proteins (Figs 2 to 4). The total abundance of PER2, not its nuclear translocation, was directly affected by *PSMD11* perturbation. It is important to note that most of the previous studies focused on the total mass of each protein in the cell. Here, we analyzed the spatiotemporal regulation of the proteins in the NRON complex—the carrier machinery. Our study of the NRON complex provides a more nuanced understanding of circadian post-translational regulation and function.

**Fig 5:**
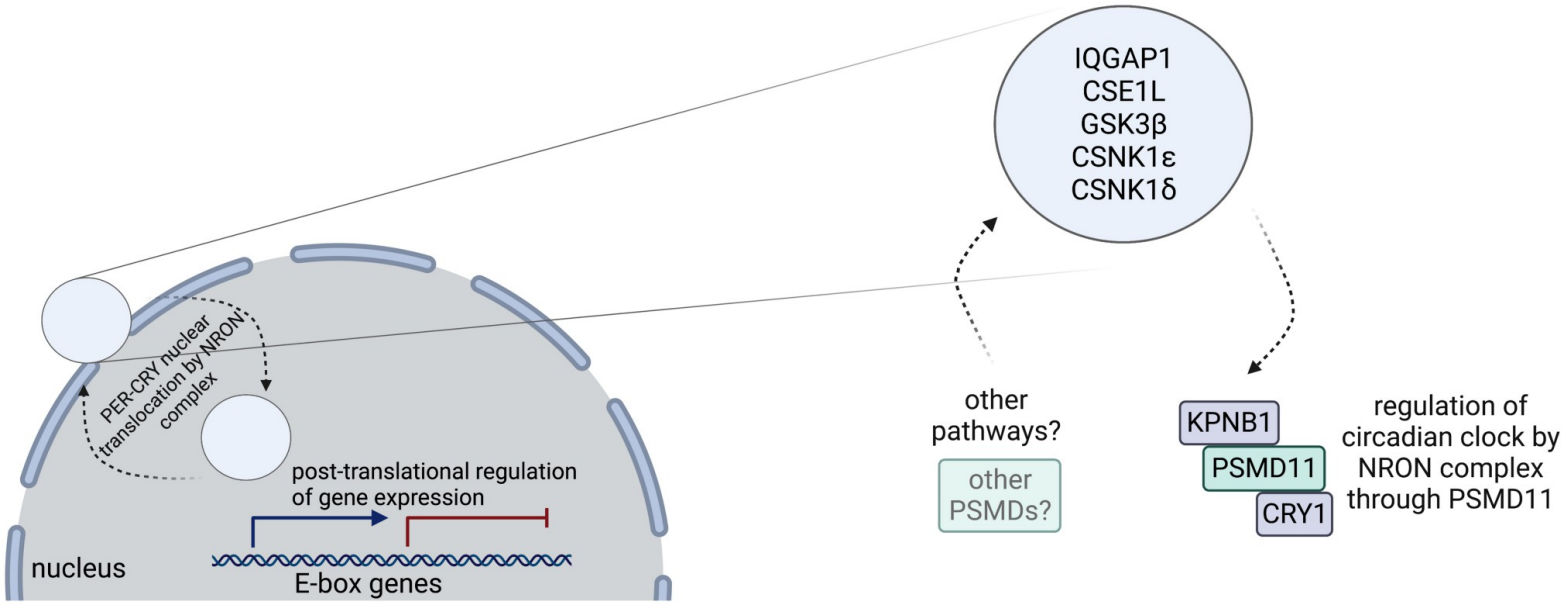
NRON complex regulates circadian clock through PSMD11 and has a potential to regulate other pathways. A model to explain the role of the NRON complex at the perinuclear area to transfer proteins between cytoplasm and nucleus. The complex is used by circadian clock and NFAT pathways and potentially is used by different pathways as well. PSMD11 controls the regulation of circadian clock through the complex, other PSMD proteins are suspected to control other pathways as a part of the complex. Created with BioRender.com.

We realized that even though PSMD4 and PSMD12 are also enriched in the NRON complex (S3G Fig), they are not required for circadian clock function. We suspect that other PSMDs that take part in the NRON complex might be regulating other pathways e.g. Wnt/*β*-catenin or hedgehog. These findings support a model (Fig 5) in which this NRON complex is located at the perinuclear region of the cell, uniquely positioned for regulating the cytoplasmic to nuclear translocation in the circadian clock, NFAT, and potentially other pathways. Presence of multiple PSMD proteins, unique positioning, and regulating more than one pathway increases the chances for the complex to control different pathways like Wnt/*β*-catenin and/or hedgehog pathways. To clarify the physical interactions between the NRON complex and these other pathways, further studies are needed.

## Experimental procedures

### Cell culture experiments

The U2 OS cell line has been used as a model cell line to detect mammalian circadian clocks [20]. This cell line was shown to demonstrate similar circadian phenotypes with animal behavioral assays [22]. Considering these previous works, we used U2 OS cells to understand time and cytosolic/nuclear aspect regulation of the complex. Our lab generated U2 OS cells with *Per2*:*dLuc and Bmal1*:*dluc* reporters with the U2 OS cell line obtained from the American Type Culture Collection (ATCC).

### siRNA experiments

The siRNA libraries used in the primary and secondary screens were purchased from QIAGEN. siRNAs used in the dose experiment are listed in S1 Table and were used as pools. Thermo RNAiMax was used to perform siRNA knockdown experiments (1 picomole per 35-mm dish) by following the vendor’s instructions. Lumicycle 32 was used to incubate the cells and measure the luminescence. WaveClock algorithm was used to determine the circadian parameters by using the luminescence recordings [21]. We used Perkin Elmer ATPlite Luminescence Assay System to detect the cell death after the siRNA treatment by following the instructions.

### Pull down experiments

To overexpress PSMD proteins to perform the pull down experiment, we purchased V5 tagged PSMD4, PSMD11, and PSMD12 from DNASU Plasmid Repository. On Day 0, U2 OS *Per2*:*dLuc* cells are seeded to have 80% confluency on the next day. On Day 1, 8 ug plasmids are transfected to 10-cm dishes, and cells are collected on Day 3. Pull down experiment was performed with Chromotek V5-Trap Agarose Kit.

### Cellular circadian clocks detection

U2 OS *Per2*:*dLuc* cells were transfected with the interested siRNAs and luminescence imaging was performed in LumiCycle32 from Actimetrics. Circadian parameters were analyzed with the WaveClock algorithm through R.

### Cytosolic-nuclear fractionation and Western blot

To investigate the temporal regulation of the NRON complex, we synchronized U2 OS cells with dexamethasone, and incubated them for 24 h. We *freshly* processed all the collected cells every 6 h and separated nuclear and cytoplasmic proteins. Small amount of cell pellets was separated to isolate whole cell proteins prior to cytoplasmic/nuclear fractionation. We first resuspended the cell pellets in the cytosolic lysis buffer (10 mM HEPES pH 7.9, 10 mM KCl, 0.1 mM EDTA, 0.05% NP40 supplemented with protease and phosphatase inhibitors) and incubated them on a rotator at 4 C. After a centrifuge at 3,000 rpm for 3 min at 4 C, we collected the supernatant and performed a second centrifuge to collect the cytosolic proteins. We washed the pellet once with the cytosolic lysis buffer and then resuspended it in the nuclear lysis buffer (20 mM HEPES pH 7.9, 0.4 M NaCl, 7.5% glycerol supplemented with protease and phosphatase inhibitors). We performed sonication two times 10 second with 85% power and centrifuged the lysates at 18,000 rpm for 10 min at 4 C to collect the nuclear proteins. We detected Tubulin as a cytosolic marker and Histone-H3 as nuclear marker protein in the collected samples with Western blot. Bio-Rad stain-free gels were used to visualize total protein amounts in each well for Fig 3A (S3D Fig). We used the Bio-Rad wet transfer system to transfer the proteins from the stain-free gels to Millipore PVDF membranes. Antibodies listed in S2 Table were used to detect the target proteins. Bio-Rad Clarity (Cat. No: 1705060) and Clarity Max (1705062) Western ECL reagents were used to illuminate chemiluminescence on the membranes. We performed the chemiluminescence imaging on a Bio-Rad ChemiDoc imager.

### Size exclusion chromatography

We used Jurkat cells to optimize the SEC protocol and determine the fractions which have the interested complex by following the protocol from [18]. We enriched complexes sized 440–670 kDa with Superdex 200 Increase 10/300 GL SEC column. We used GE Healthcare gel filtration Calibration Kit (Cat No. 28-4038-42) to detect the molecular weight chromatogram for the column (S3B Fig). We performed Western blot to determine the interested fractions having the NRON complex. We detected several known complex components in fractions 16 to 40 (between the void fraction and 158 kDa marker) with Western blot (S3C Fig). We concluded that fractions 22–25 are the fractions having the NRON complex. We analyzed these fractions on the further planned assay conditions.

## Supporting information

S1 Fig Supplemental information of the investigation the PSMD effect on the cellular circadian rhythms. (A) Effects of PSMD4, PSMD11, and PSMD12 are not due to cell death. *PSMD11*, *PSMD4* and *PSMD12* knockdown ATPlite cell cytotoxicity assay system results for U2 OS *Per2*:*dLuc* reporter line. (B) Stain-free loading controls for Western Blots in Fig 1D. (C), (D), (E) *PSMD11*, *PSMD4* and *PSMD12* knockdown cellular circadian profiles. Representative bioluminescence records of circadian rhythms in U2 OS *Bmal1*:*dLuc* reporter cells. n = 3 independent experiments (F) Baseline, amplitude and phase results of C-E that are calculated with WaveClock algorithm. (G) mRNA levels detected with QPCR to check the RNAi knockdown effects. mRNA levels are normalized to GAPDH. Statistical analyses were performed with unpaired t tests. Error bars represent SEM.
(TIF)

S2 Fig Protein level regulation of CRY2 and PER2 by PSMD11. (A) PER1, PER2, CRY1 and CRY2 levels are detected in control (*siNEG*) and *siNRON* samples in U2OS cells. n = 3 replicates, significancy is analyzed with unpaired t test. (B) *CRY1*, *CRY2*, and *PER2* mRNA levels are detected in control (*siNEG*) and *siPSMD11* samples in U2 OS cells. *siPSMD11* does not affect the detected mRNA levels. (C) FBXL21 mRNA levels in the samples of Fig 2B.
(TIF)

S3 Fig Enrichment and analysis of the NRON complex with size exclusion chromatography. (A) Methodology for size exclusion chromatography. Extracted and dialyzed proteins were used to enrich the NRON complex through Superdex 200 Increase 10/300 GL size exclusion chromatography column. Created with BioRender.com (B) Molecular weight chromatogram for Superdex 200 Increase 10/300 GL size exclusion chromatography column. GE Healthcare Gel Filtration Calibration HMW Kit is used to prepare the chromatogram. (C) Fraction analysis with western blot to determine the fraction containing the NRON complex components. IQGAP1, CRY1, CSNK1*ε*, and GSK3*β* are detected in the collected fractions by Western blot. (D) Stain-free loading control for the blot in Fig 3A. (E) Cytoplasmic and nuclear fractionation verification with Western blot. Tubulin and Histone-H3 are markers of cytoplasmic and nuclear fractions, respectively. (F) CRY1 in the protein lysates of cytoplasmic and nuclear fractions before the SEC experiment. (G) PSMD12 and PSMD4 levels at cytoplasmic and nuclear fractions within the NRON complex.
(ZIP)

S1 Table siRNA catalog numbers from QIAGEN. (TIF)

S2 Table Antibodies used to detect the proteins in Western blot. (TIF)

S1 Raw images Uncropped Western blot images. (PDF)

S1 File (PDF)
