# Supplementary figures and images for: PSMD11 modulates circadian clock function through PER and CRY nuclear translocation
Source: PLoS One. 2023 Mar 24;18(3):e0283463. doi: 10.1371/journal.pone.0283463 (PMC10038281; doi:10.1371/journal.pone.0283463)

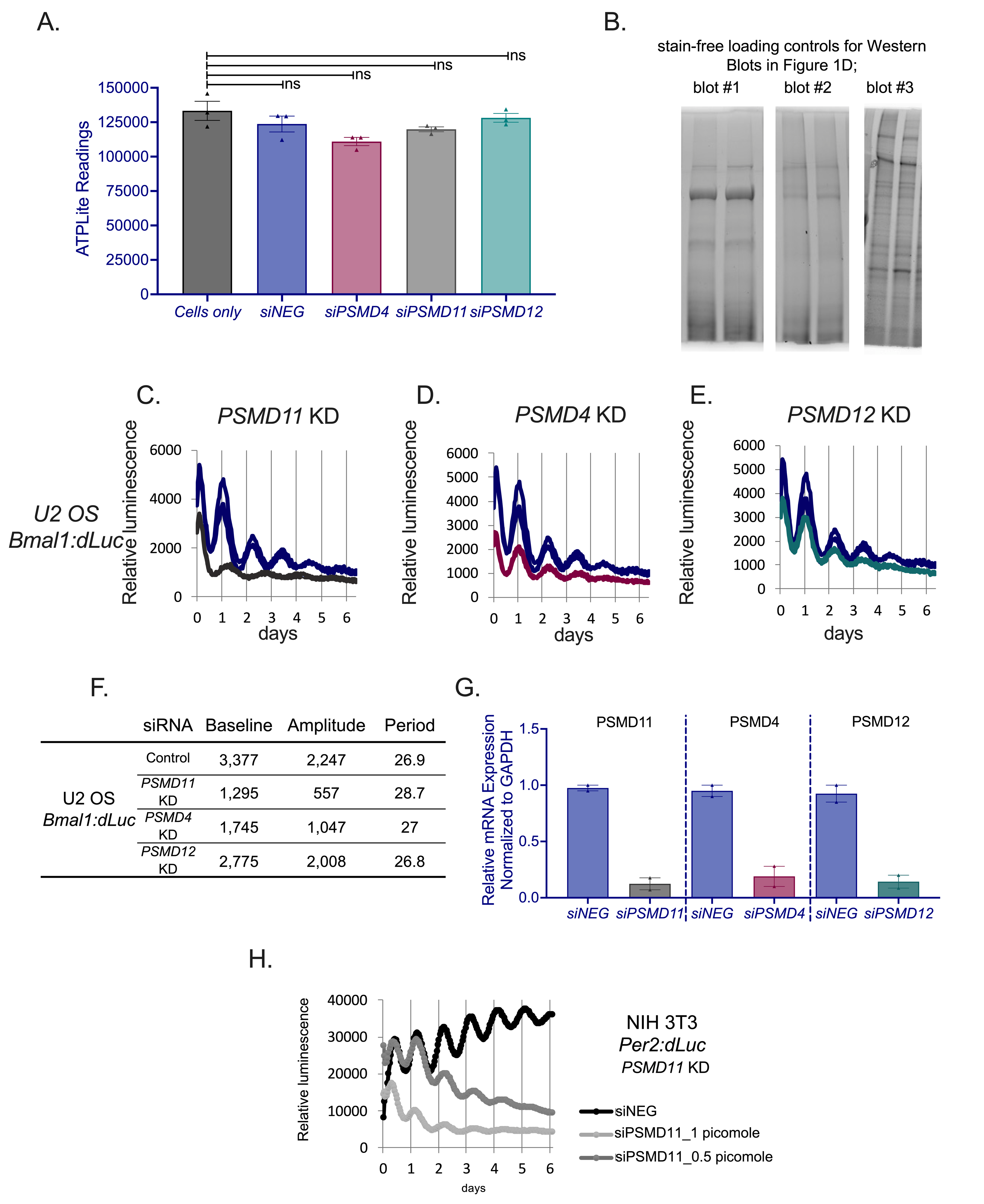

Supplement: S1 Fig — (A) Effects of PSMD4, PSMD11, and PSMD12 are not due to cell death. PSMD11, PSMD4 and PSMD12 knockdown ATPlite cell cytotoxicity assay system results for U2 OS Per2:dLuc reporter line. (B) Stain-free loading controls for Western Blots in Fig 1D. (C), (D), (E) PSMD11, PSMD4 and PSMD12 knockdown cellular circadian profiles. Representative bioluminescence records of circadian rhythms in U2 OS Bmal1:dLuc reporter cells. n = 3 independent experiments (F) Baseline, amplitude and phase results of C-E that are calculated with WaveClock algorithm. (G) mRNA levels detected with QPCR to check the RNAi knockdown effects. mRNA levels are normalized to GAPDH. Statistical analyses were performed with unpaired t tests. Error bars represent SEM. (TIF) [file pone.0283463.s001.tif]

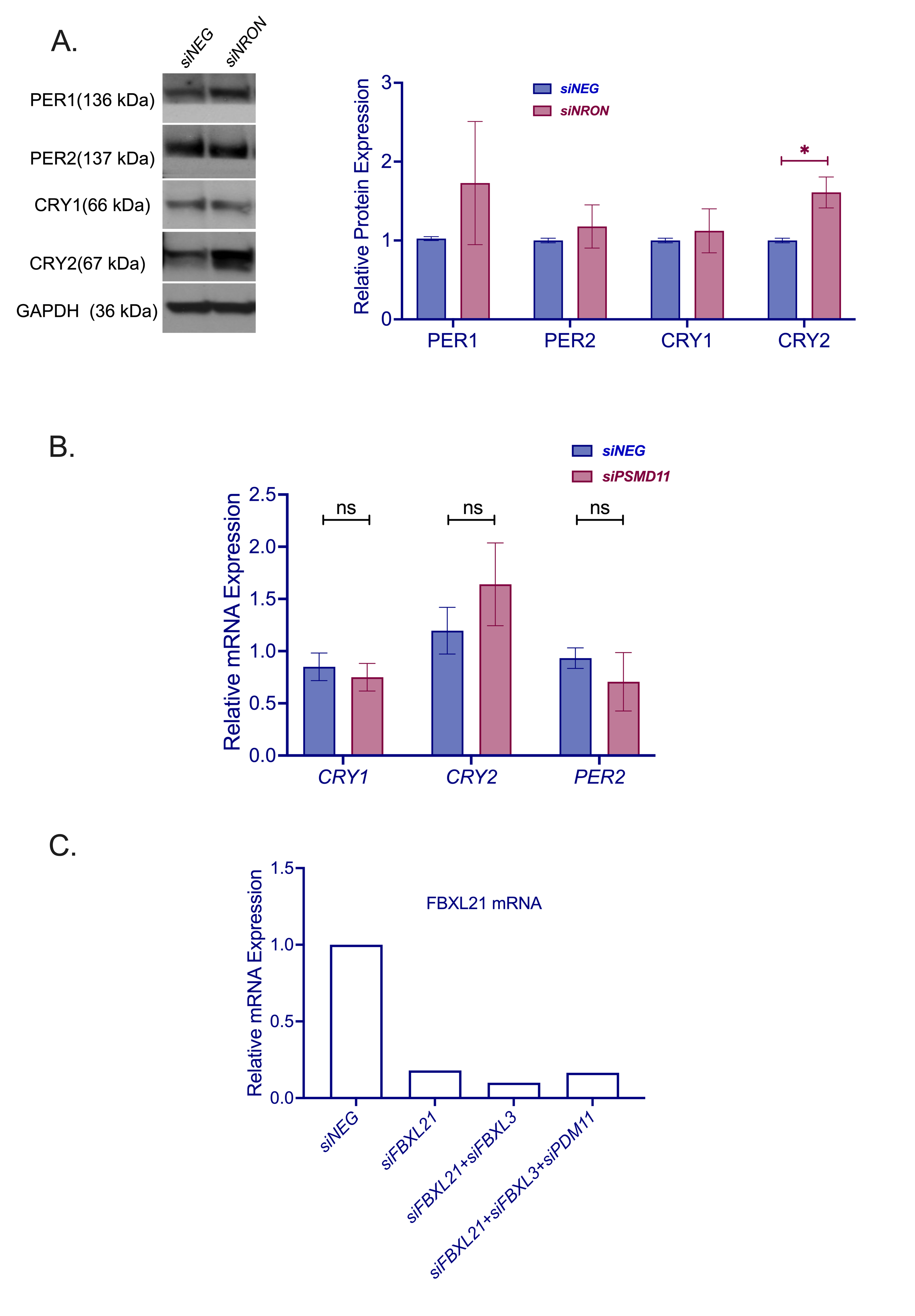

Supplement: S2 Fig — (A) PER1, PER2, CRY1 and CRY2 levels are detected in control (siNEG) and siNRON samples in U2OS cells. n = 3 replicates, significancy is analyzed with unpaired t test. (B) CRY1, CRY2, and PER2 mRNA levels are detected in control (siNEG) and siPSMD11 samples in U2 OS cells. siPSMD11 does not affect the detected mRNA levels. (C) FBXL21 mRNA levels in the samples of Fig 2B. (TIF) [file pone.0283463.s002.tif]

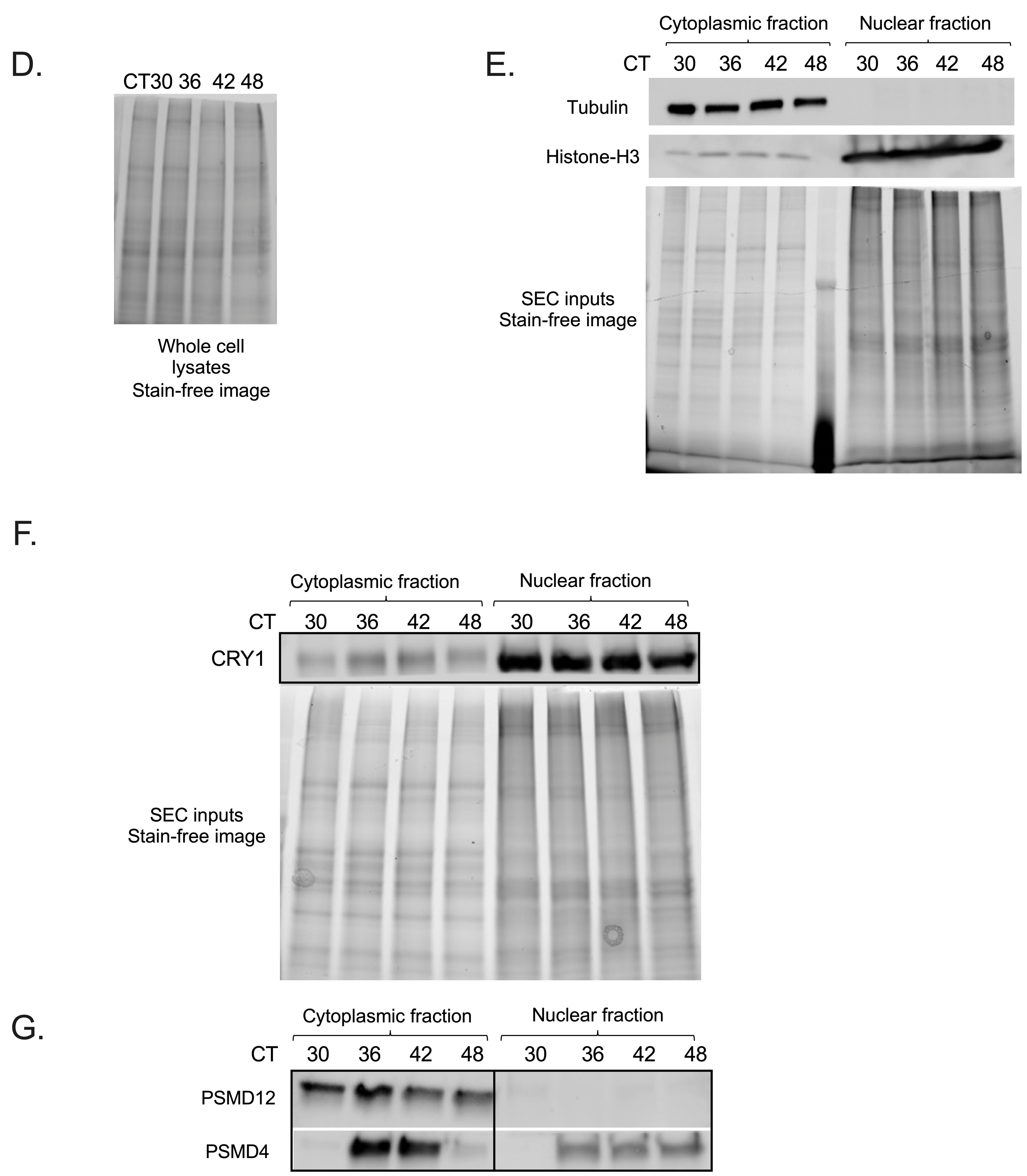

Supplement: S3 Fig — (A) Methodology for size exclusion chromatography. Extracted and dialyzed proteins were used to enrich the NRON complex through Superdex 200 Increase 10/300 GL size exclusion chromatography column. Created with BioRender.com (B) Molecular weight chromatogram for Superdex 200 Increase 10/300 GL size exclusion chromatography column. GE Healthcare Gel Filtration Calibration HMW Kit is used to prepare the chromatogram. (C) Fraction analysis with western blot to determine the fraction containing the NRON complex components. IQGAP1, CRY1, CSNK1ε, and GSK3β are detected in the collected fractions by Western blot. (D) Stain-free loading control for the blot in Fig 3A. (E) Cytoplasmic and nuclear fractionation verification with Western blot. Tubulin and Histone-H3 are markers of cytoplasmic and nuclear fractions, respectively. (F) CRY1 in the protein lysates of cytoplasmic and nuclear fractions before the SEC experiment. (G) PSMD12 and PSMD4 levels at cytoplasmic and nuclear fractions within the NRON complex. (ZIP) [file pone.0283463.s003.zip › S3_Fig_2.tif]

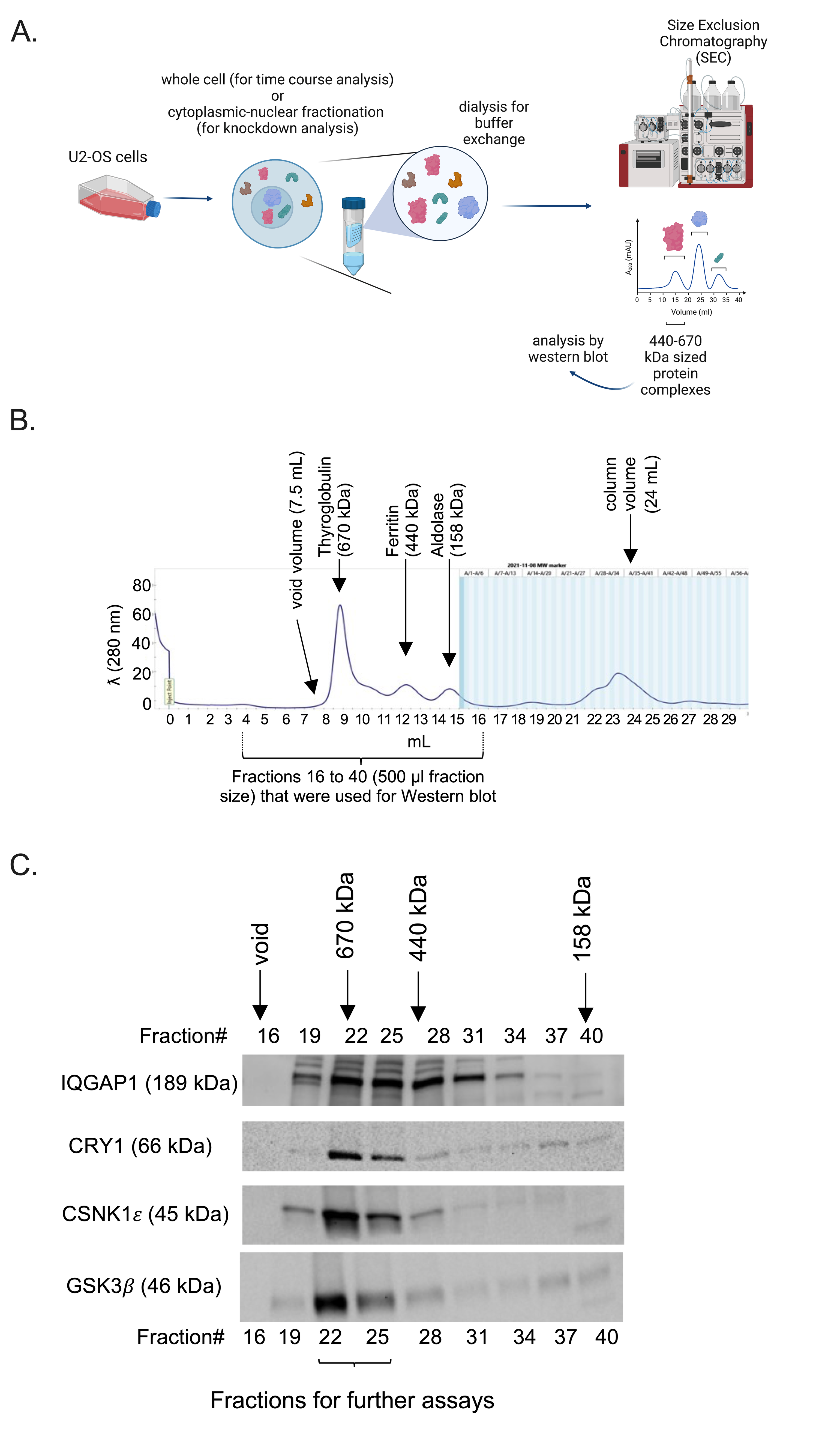

Supplement: S3 Fig — (A) Methodology for size exclusion chromatography. Extracted and dialyzed proteins were used to enrich the NRON complex through Superdex 200 Increase 10/300 GL size exclusion chromatography column. Created with BioRender.com (B) Molecular weight chromatogram for Superdex 200 Increase 10/300 GL size exclusion chromatography column. GE Healthcare Gel Filtration Calibration HMW Kit is used to prepare the chromatogram. (C) Fraction analysis with western blot to determine the fraction containing the NRON complex components. IQGAP1, CRY1, CSNK1ε, and GSK3β are detected in the collected fractions by Western blot. (D) Stain-free loading control for the blot in Fig 3A. (E) Cytoplasmic and nuclear fractionation verification with Western blot. Tubulin and Histone-H3 are markers of cytoplasmic and nuclear fractions, respectively. (F) CRY1 in the protein lysates of cytoplasmic and nuclear fractions before the SEC experiment. (G) PSMD12 and PSMD4 levels at cytoplasmic and nuclear fractions within the NRON complex. (ZIP) [file pone.0283463.s003.zip › S3_Fig_1.tif]

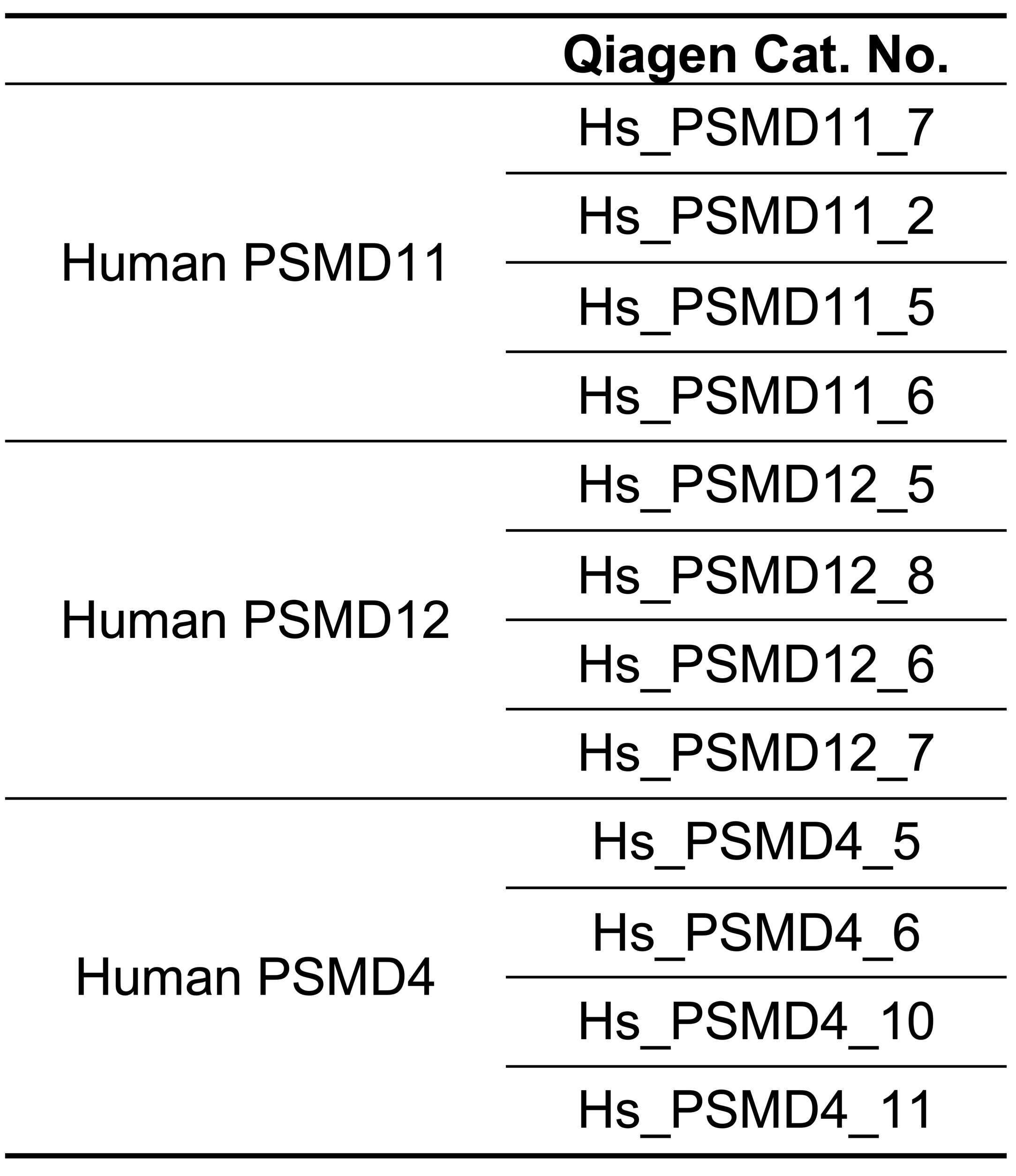

Supplement: S1 Table — (TIF) [file pone.0283463.s004.tif]

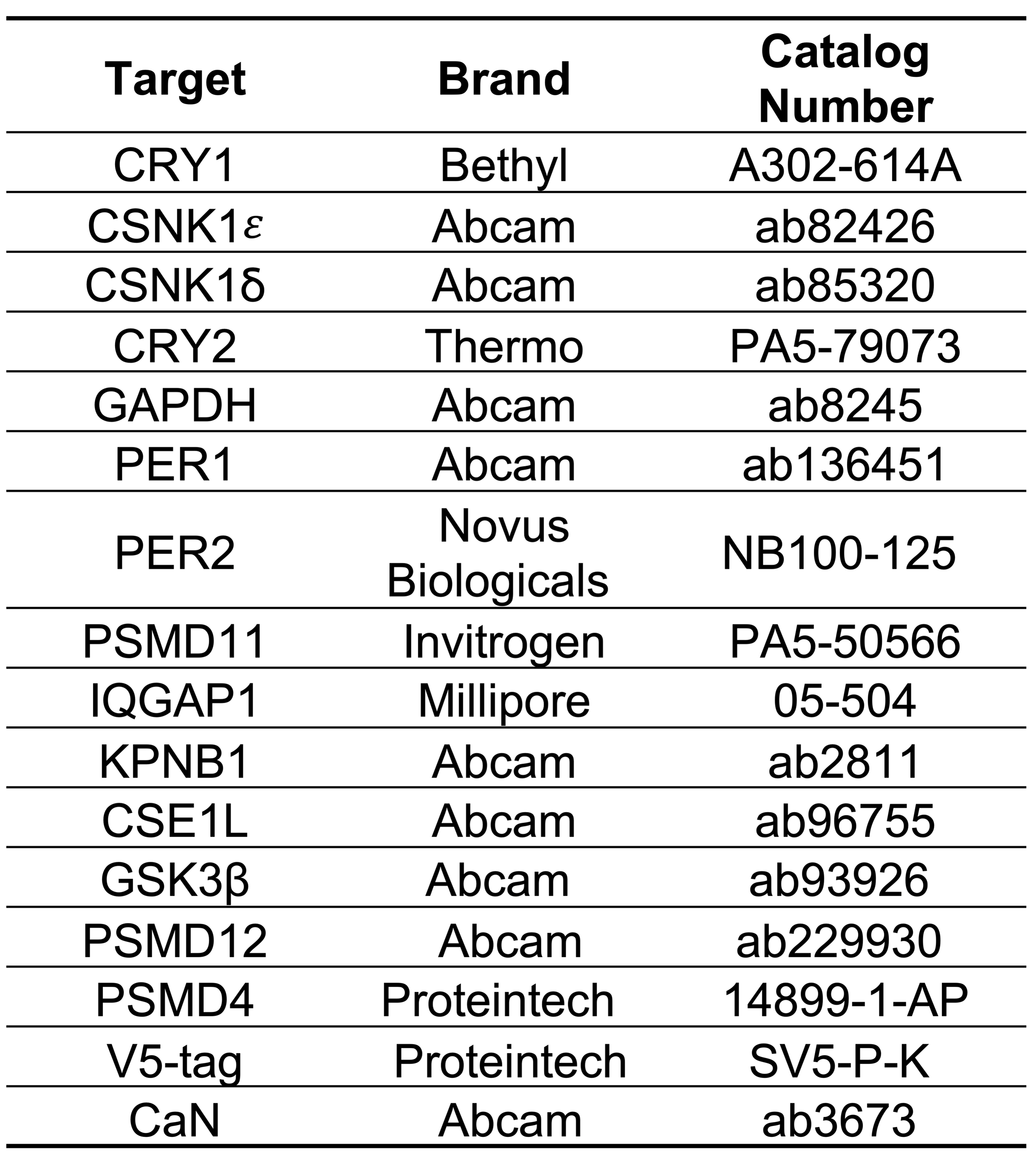

Supplement: S2 Table — (TIF) [file pone.0283463.s005.tif]
