# Supplementary material for: PSMD11 modulates circadian clock function through PER and CRY nuclear translocation
Source: PLoS One. 2023 Mar 24;18(3):e0283463. doi: 10.1371/journal.pone.0283463 (PMC10038281; doi:10.1371/journal.pone.0283463)
Supplement: S1 Raw images — (PDF) [file pone.0283463.s006.pdf]

Figure 1E

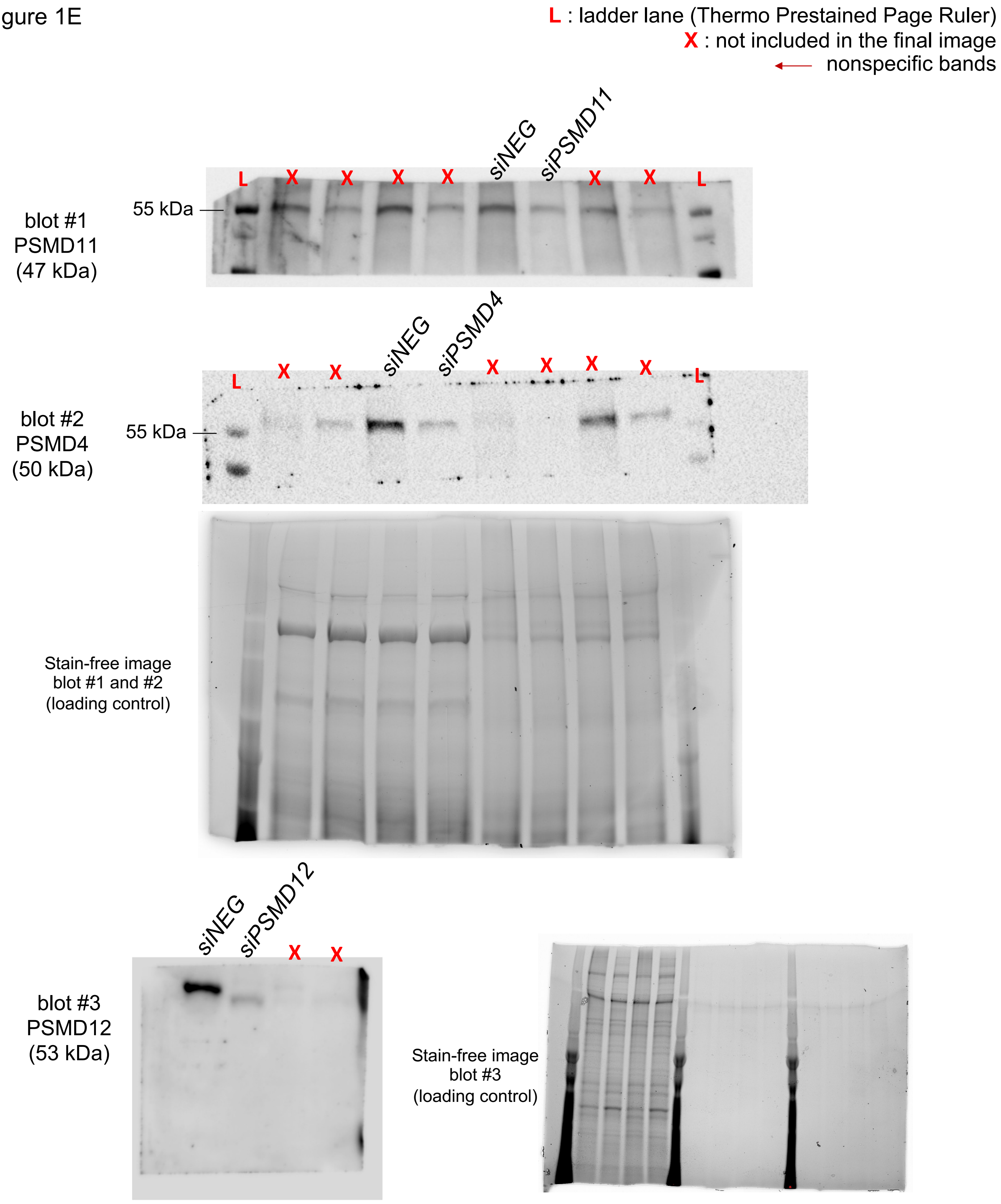
L : ladder lane (Thermo Prestained Page Ruler)
X : not included in the final image
nonspecific bands
blot #1
PSMD11
(47 kDa)
55 kDa
L
X
X
X
X
siNEG
siPSMD11
X
X
L
blot #2
PSMD4
(50 kDa)
55 kDa
L
X
X
siNEG
siPSMD4
X
X
X
X
L
Stain-free image
blot #1 and #2
(loading control)
blot #3
PSMD12
(53 kDa)
siNEG
siPSMD12
X
X
Stain-free image
blot #3
(loading control)

Figure 2A-three replicates

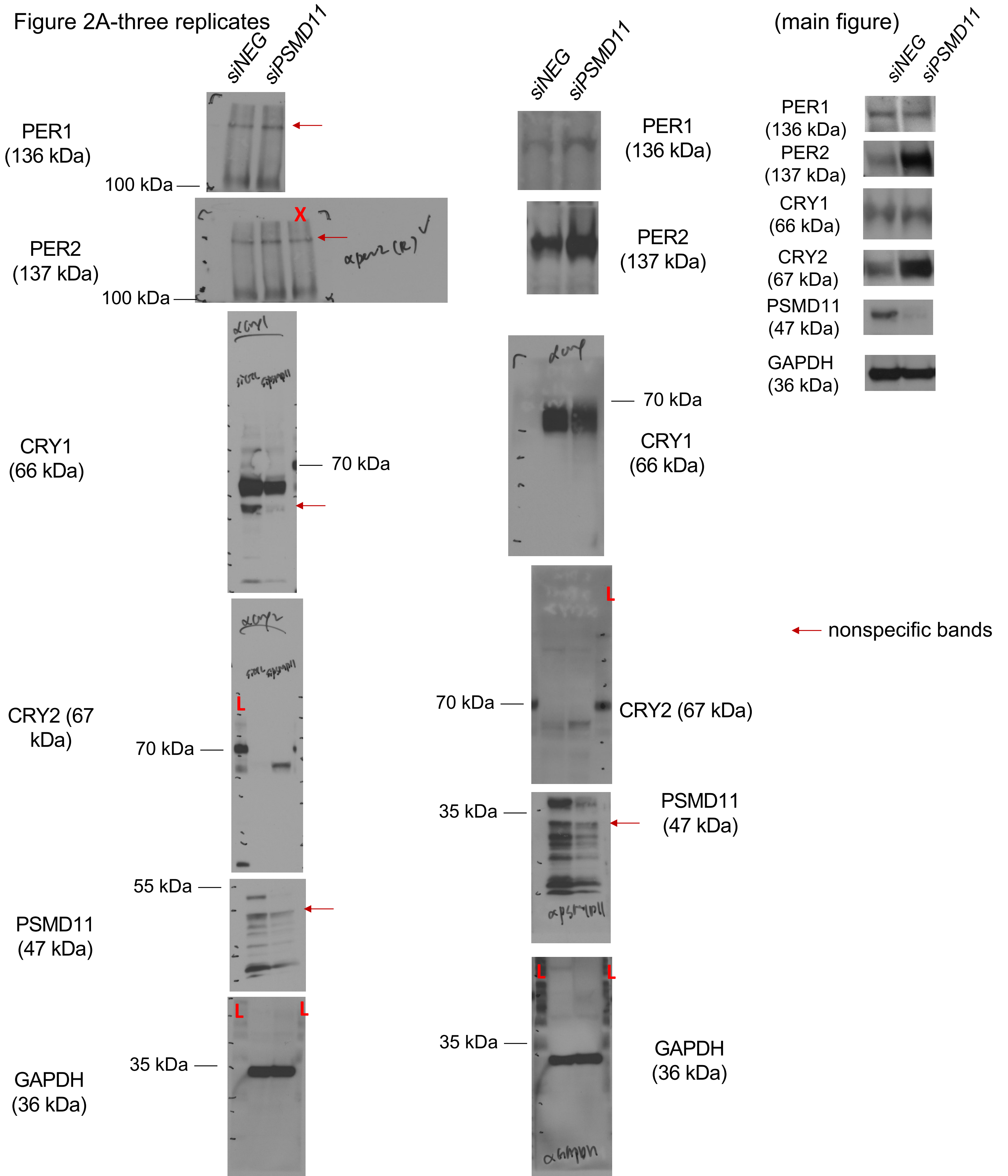

Figure 2B

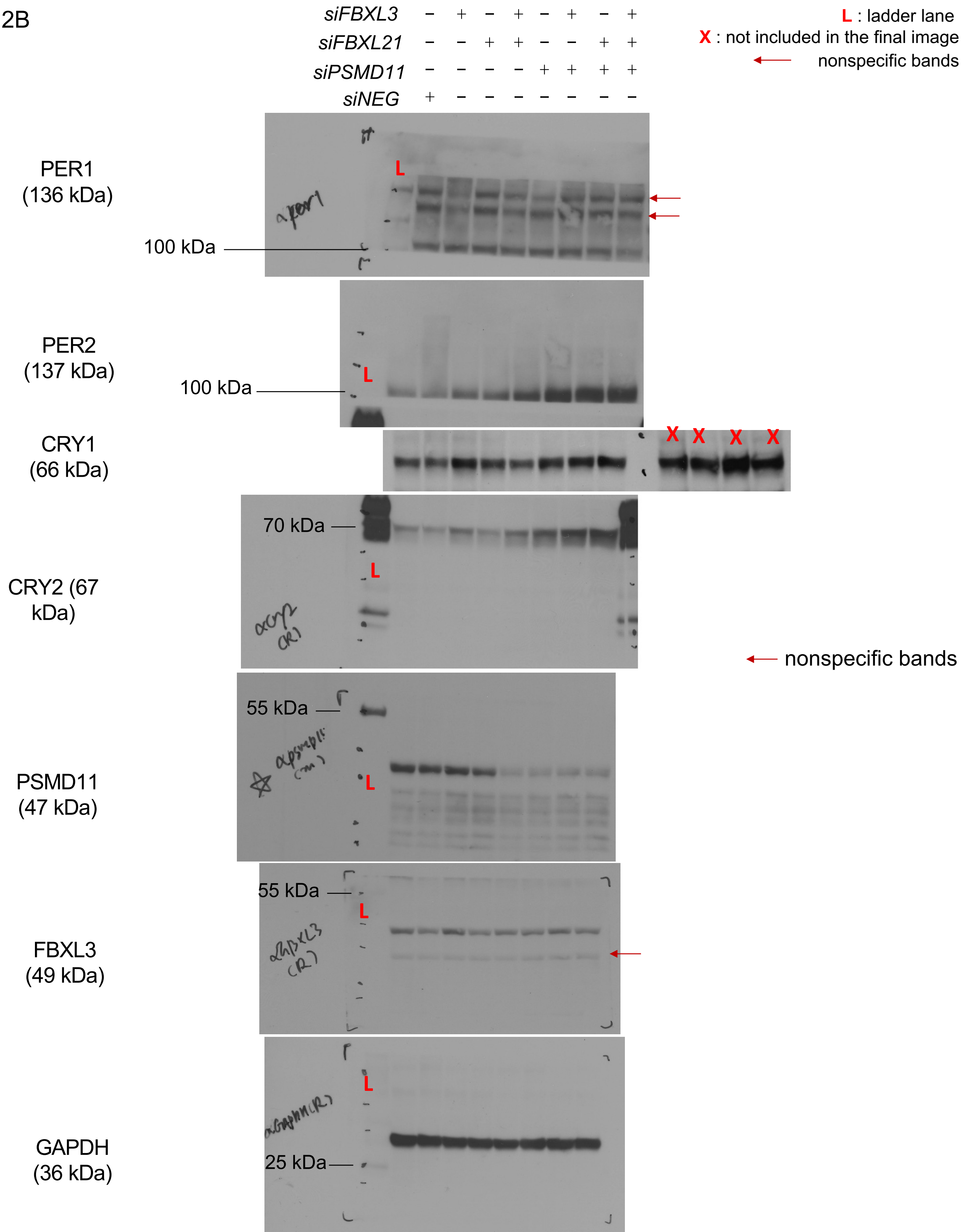
siFBXL3 − + − + − + − +
siFBXL21 − − + + − − + +
siPSMD11 − − − − + + + +
siNEG + − − − − − − −
L : ladder lane
X : not included in the final image
nonspecific bands
PER1 (136 kDa)
100 kDa
PER2 (137 kDa)
100 kDa
CRY1 (66 kDa)
X X X X
CRY2 (67 kDa)
70 kDa
nonspecific bands
PSMD11 (47 kDa)
55 kDa
FBXL3 (49 kDa)
55 kDa
GAPDH (36 kDa)
25 kDa
L

Figure 3A-three replicates

L : ladder lane
X : not included in the final image
← nonspecific bands

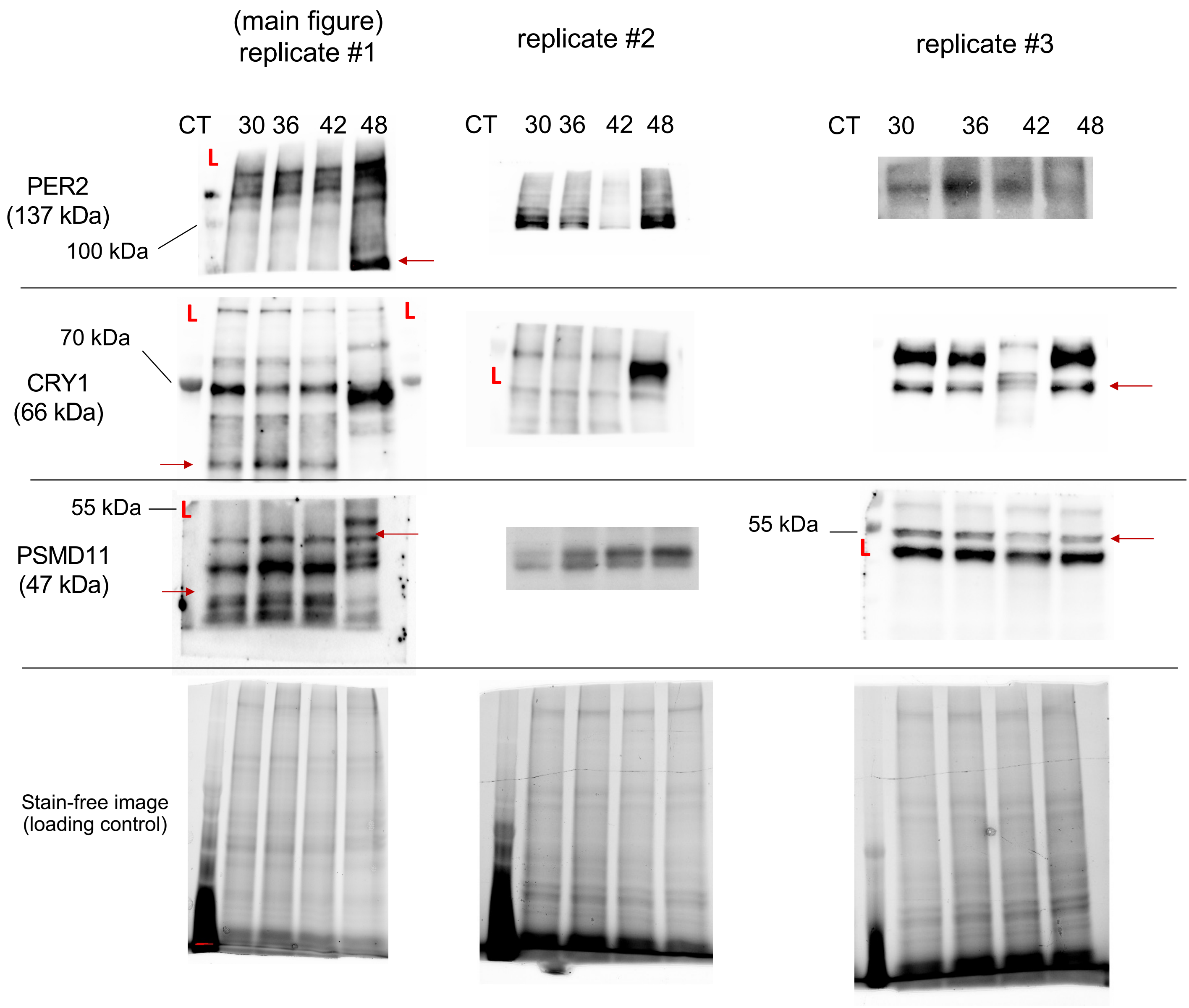

← nonspecific bands

Figure 3B-replicate #1 (main figure)

L : ladder lane
X : not included in the final image
← nonspecific bands

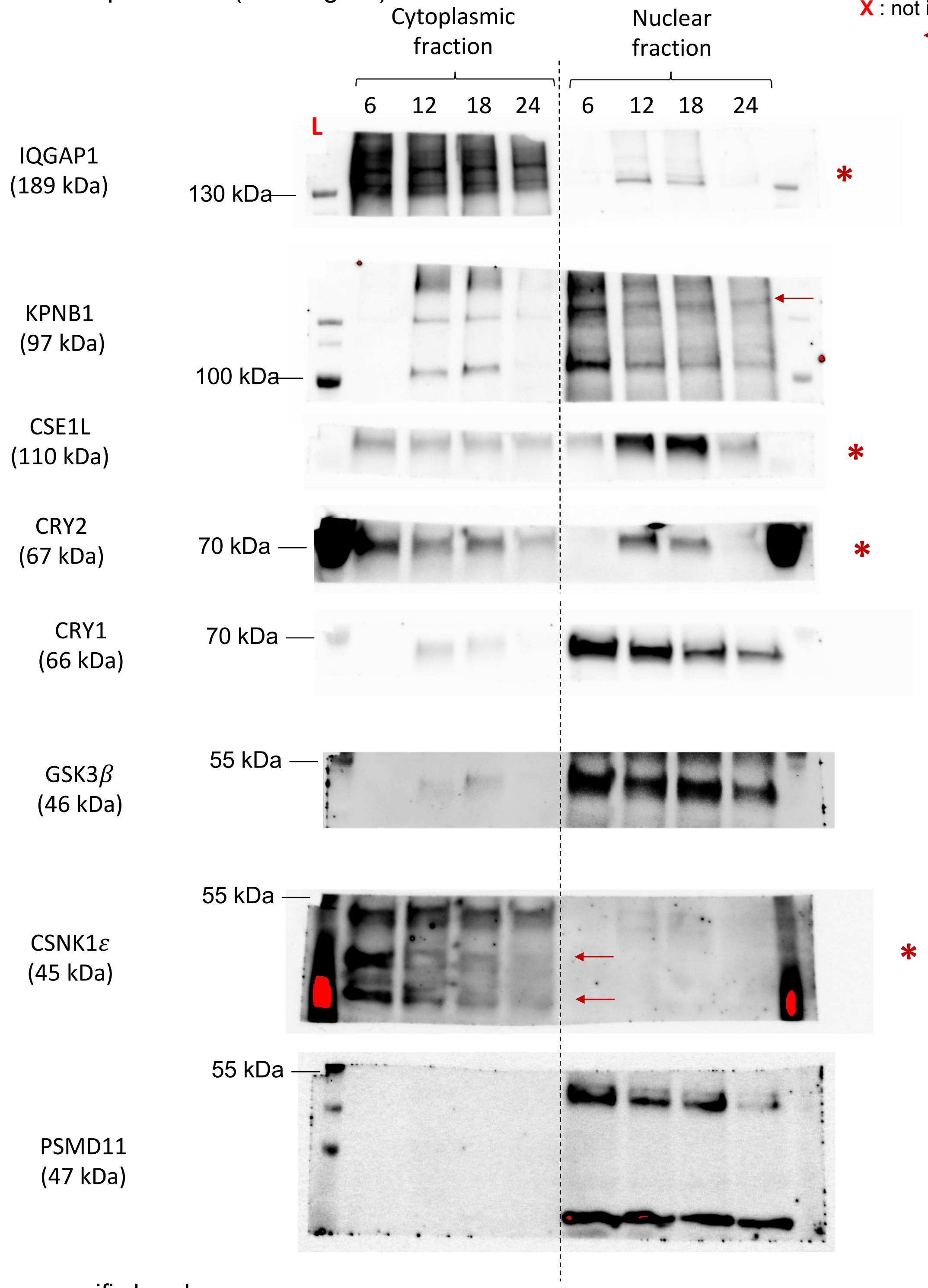

← nonspecific bands

***cytoplasmic and nuclear fractions are flipped. Same images are cut and pasted together for the final figure**

Figure 3B- replicate #2

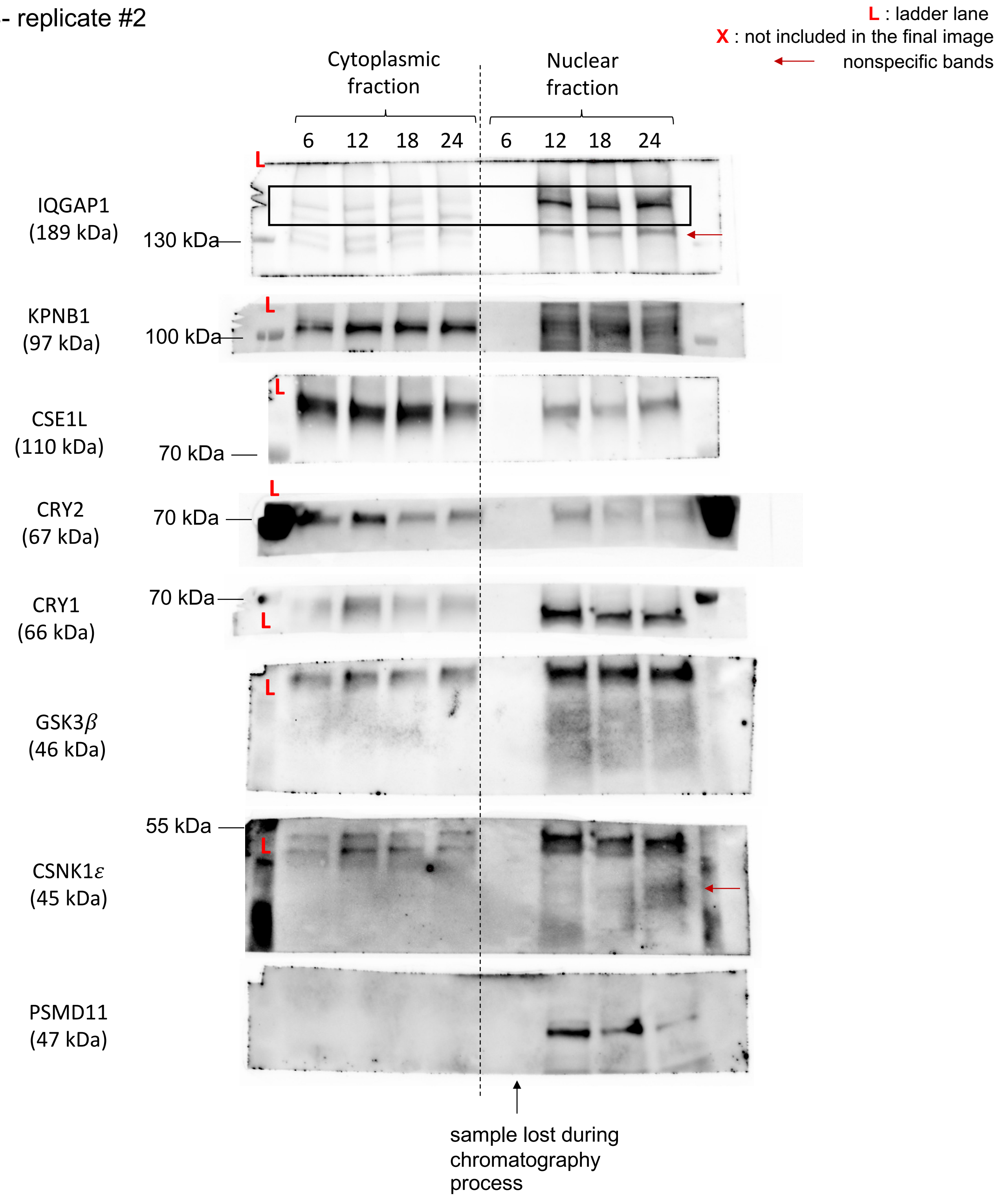

nonspecific bands

Figure 4A-replicate #1 (main figure)

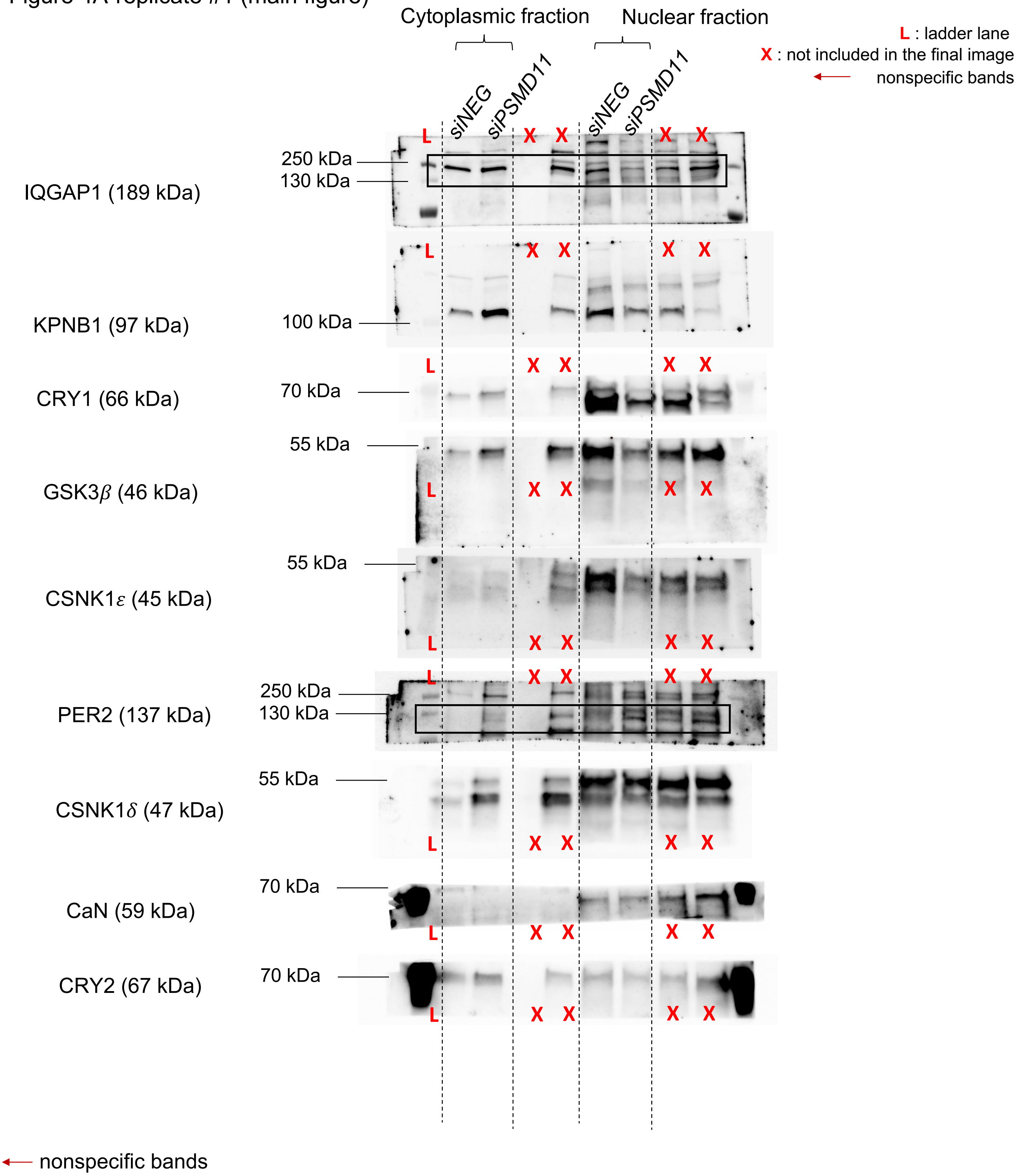

Figure 4A-replicate #2

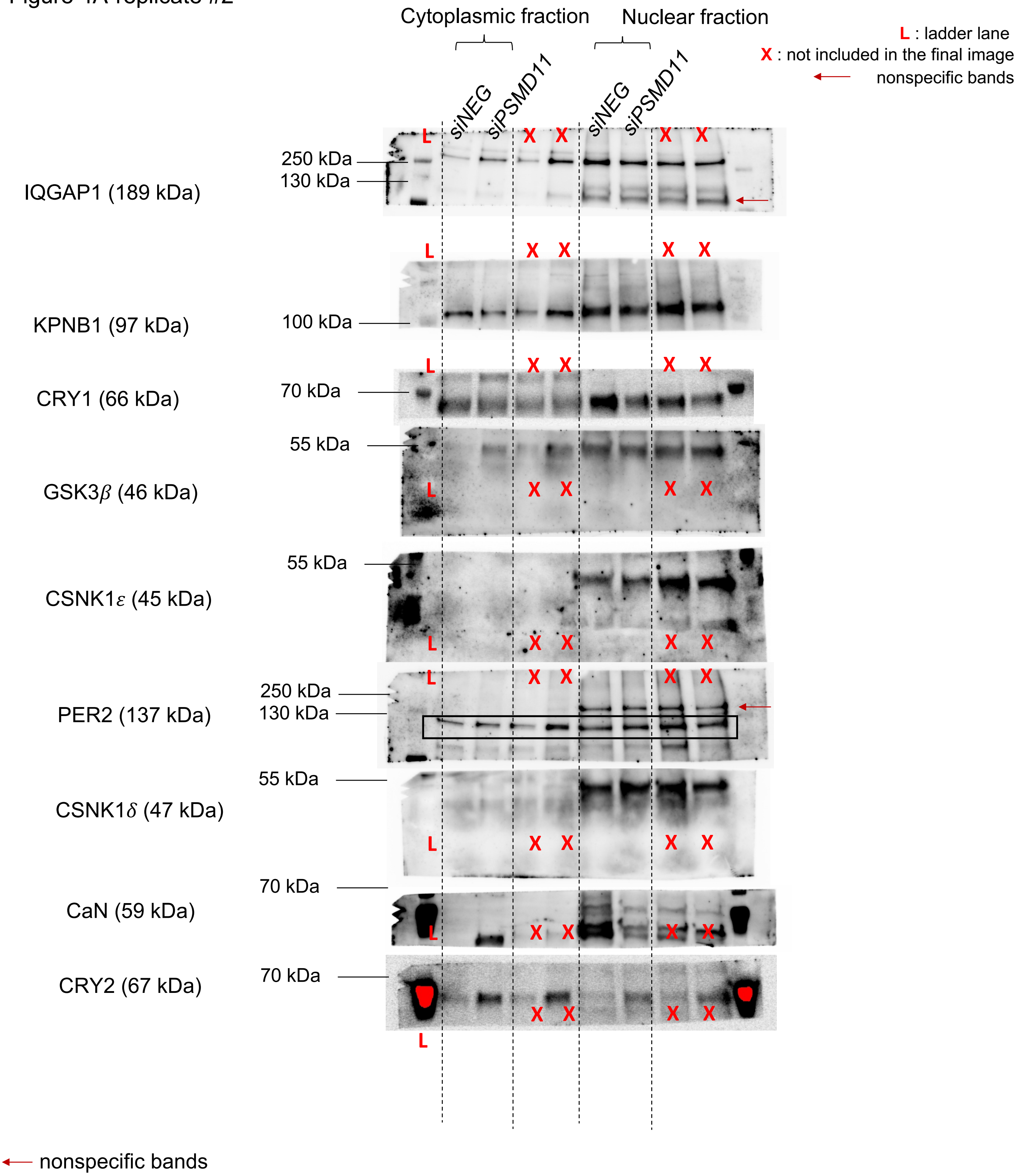

Figure 4A-replicate #3

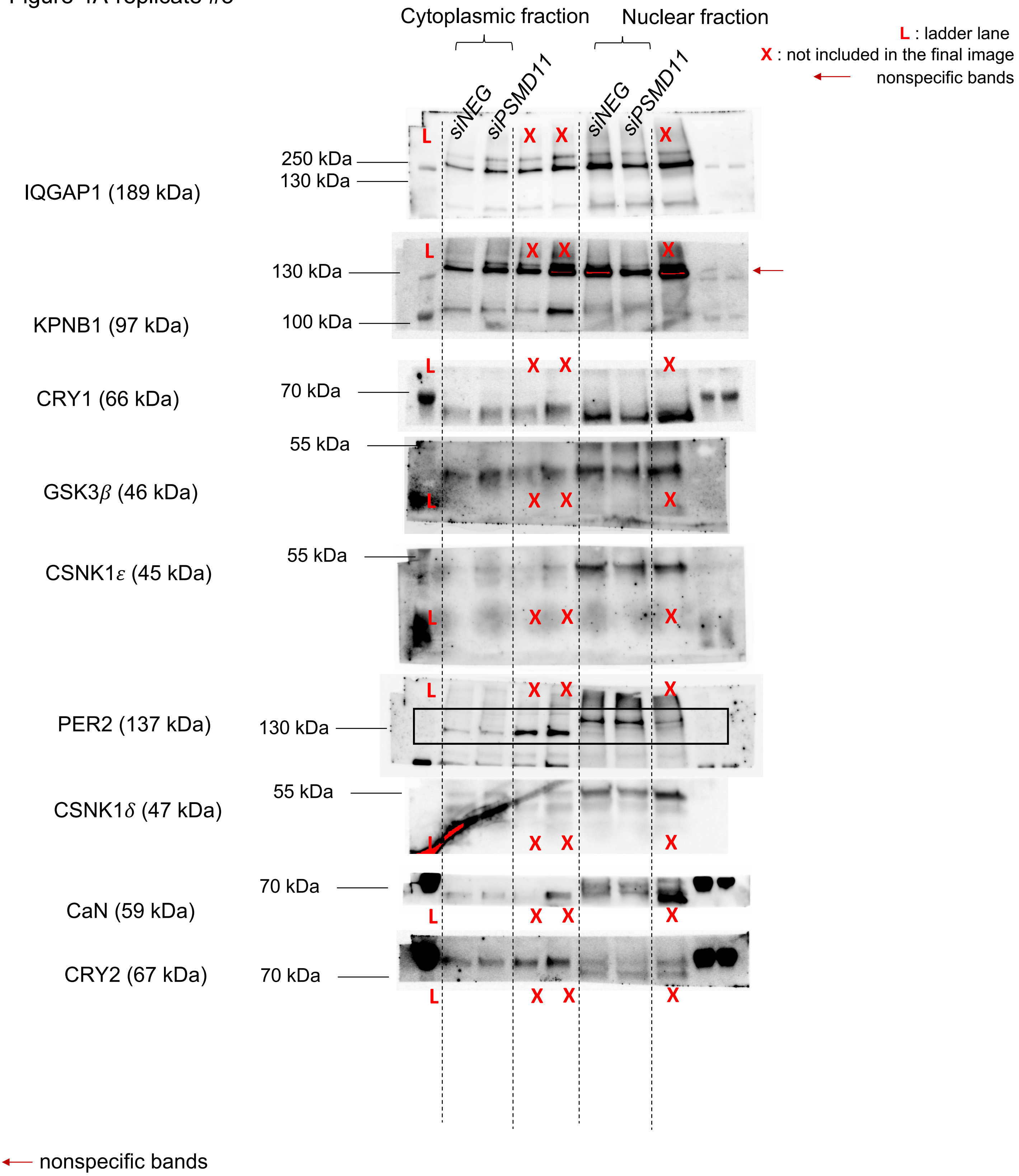

Figure 4C

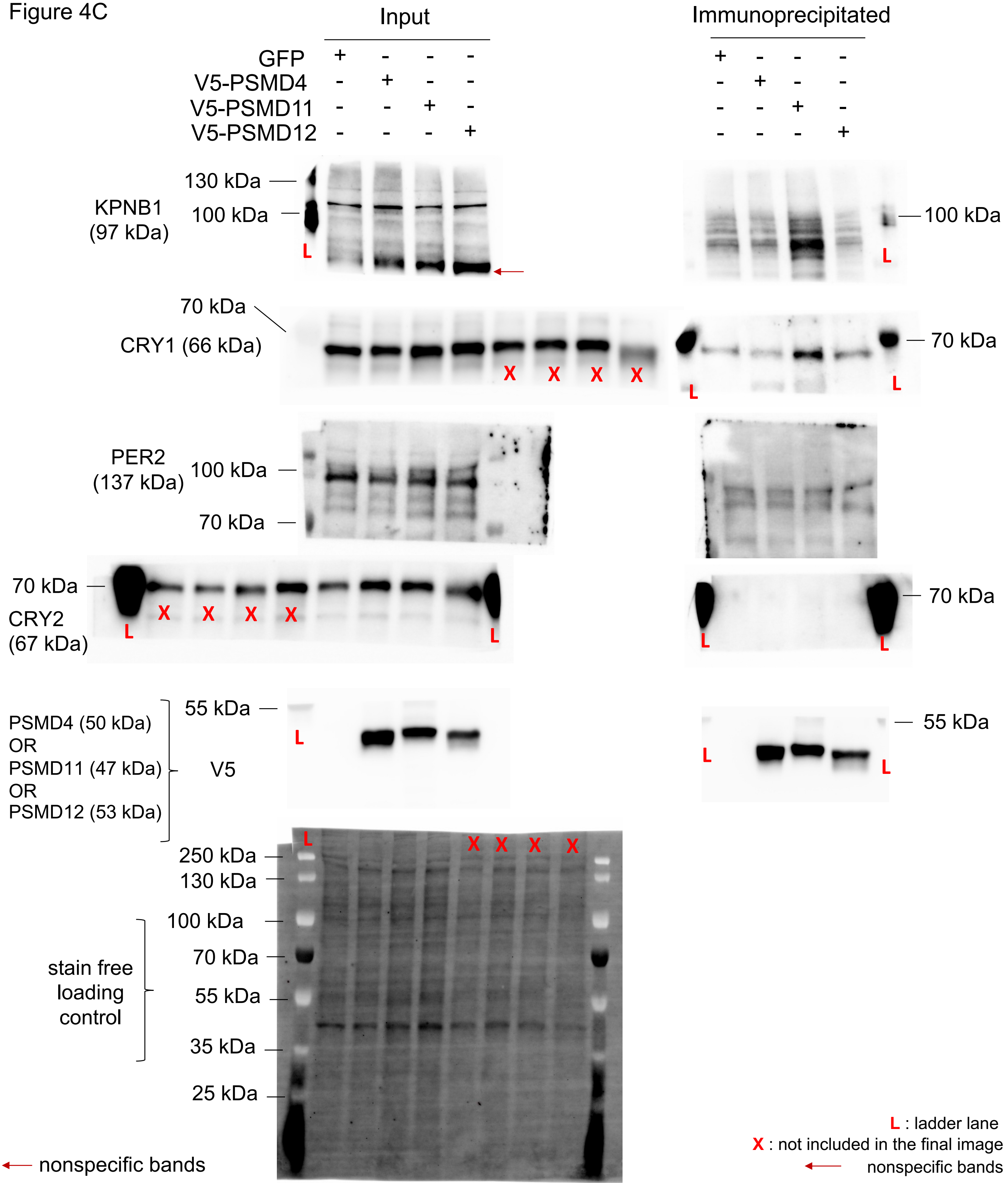
Input
Immunoprecipitated
GFP
V5-PSMD4
V5-PSMD11
V5-PSMD12
KPNB1
(97 kDa)
130 kDa
100 kDa
CRY1 (66 kDa)
70 kDa
PER2
(137 kDa)
100 kDa
70 kDa
CRY2
(67 kDa)
70 kDa
PSMD4 (50 kDa)
OR
PSMD11 (47 kDa)
OR
PSMD12 (53 kDa)
V5
55 kDa
stain free
loading
control
250 kDa
130 kDa
100 kDa
70 kDa
55 kDa
35 kDa
25 kDa
nonspecific bands
L : ladder lane
X : not included in the final image
nonspecific bands

Figure 4C-replicate #2

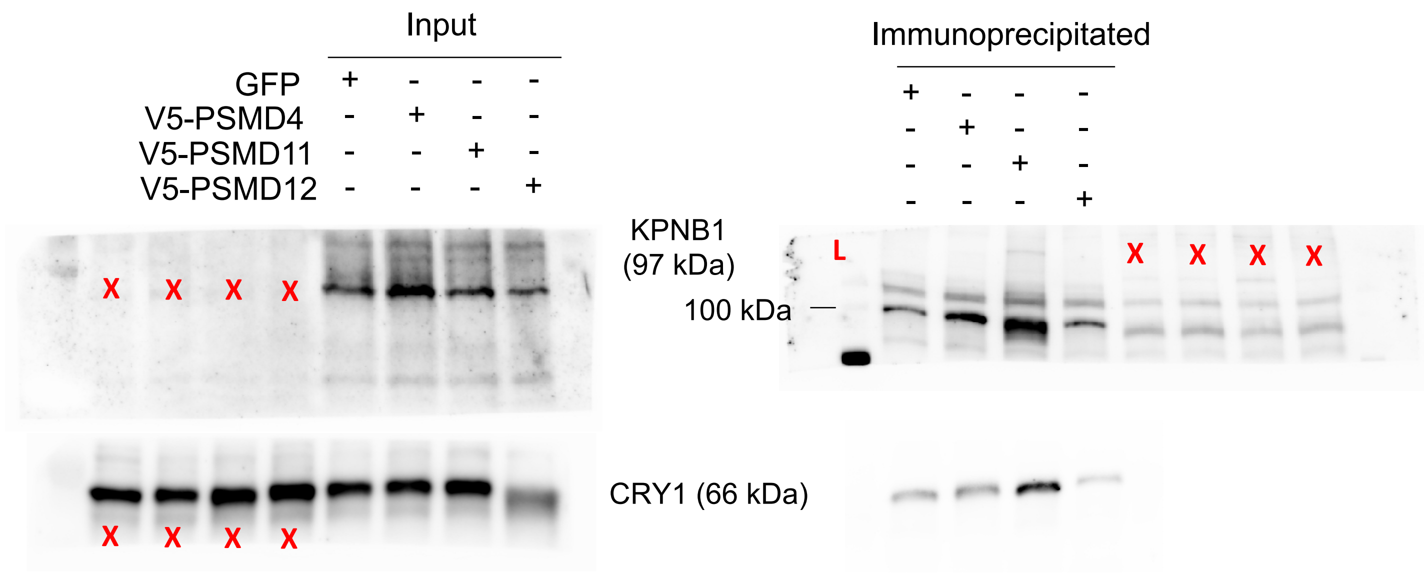

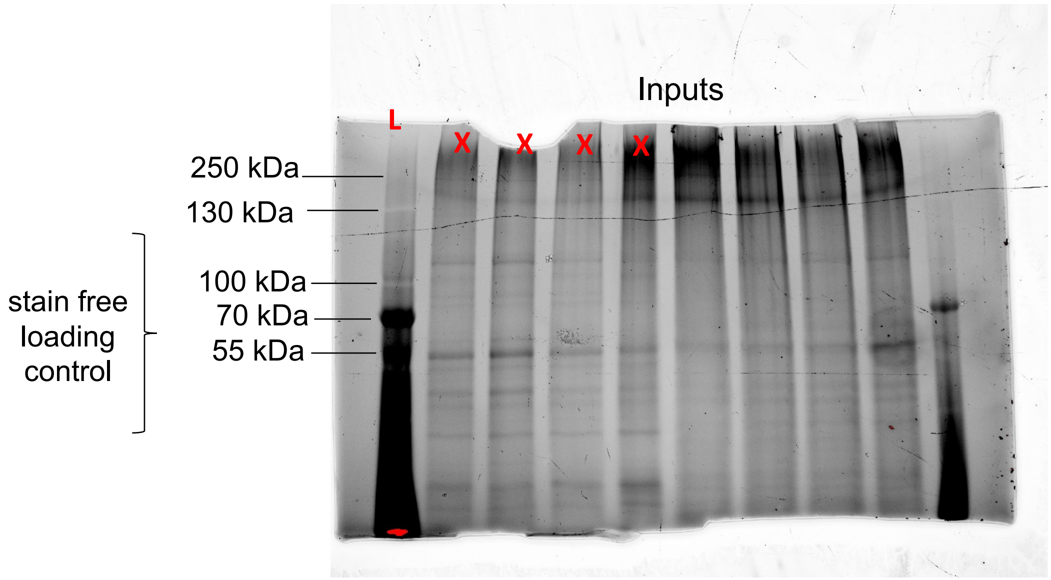

L : ladder lane
X : not included in the final image
nonspecific bands
