# Supplementary material for: PSMD11 modulates circadian clock function through PER and CRY nuclear translocation
Source: PLoS One. 2023 Mar 24;18(3):e0283463. doi: 10.1371/journal.pone.0283463 (PMC10038281; doi:10.1371/journal.pone.0283463)
Supplement: S1 File — (PDF) [file pone.0283463.s007.pdf]

Fig 1A-B-C						
Time	Neg si_P	Neg si_P	Neg si_P	PSMD11	PSMD11	PSMD11
0	9483	10314	10187	27954	26682	26056
0.04	10846	12197	11649	25263	25109	24333
0.08	11793	13184	12703	22676	22625	21799
0.11	12922	14314	14110	21053	21297	20748
0.15	14435	15842	15396	21056	21099	20461
0.19	15437	16885	16847	20878	21172	20599
0.23	16159	17981	18016	21454	21814	21279
0.26	17024	18721	18756	22358	22437	21698
0.3	17742	19301	19440	23127	23351	22491
0.34	18314	19865	19944	23972	24233	23068
0.38	18530	20248	20406	24602	24747	23730
0.41	19233	20666	20747	25137	25038	24120
0.45	19244	20405	20600	25124	25253	24645
0.49	18803	20215	20653	25032	25290	24683
0.53	18136	19563	20248	24486	24376	23708
0.57	17605	18872	19415	23675	23683	23157
0.6	16845	18081	18284	22473	22587	22086
0.64	15706	17055	17213	20873	20695	20841
0.68	14738	15465	15796	19295	19378	18912
0.72	13173	14307	14629	18004	17445	17203
0.75	12231	13060	13250	16064	15906	15788
0.79	10945	11729	11899	14348	14067	14094
0.83	9914	10604	10740	12712	12600	12627
0.87	8918	9327	9581	11292	11166	11305
0.9	8144	8719	8621	10162	10002	9993
0.94	7393	7901	8071	9401	9155	9072
0.98	6964	7566	7386	8500	8546	8551
1.02	6761	7379	7249	8158	8058	8016
1.06	6665	7272	7200	7777	7787	7827
1.09	6649	7483	7354	7777	7740	7757
1.13	6881	7765	7660	7970	8037	8130
1.17	7292	8252	8093	8454	8427	8475
1.21	7875	8942	8847	9010	8956	8835
1.24	8340	9441	9346	9481	9499	9419
1.28	8942	10160	10054	10008	10087	9866
1.32	9485	10696	10366	10550	10501	10426
1.36	9758	10995	10992	11092	11136	10716
1.39	10089	11337	11210	11508	11245	11120
1.43	10307	11717	11524	11492	11591	11377

1.47	10389	11856	11669	11658	11903	11552
1.51	10587	11860	11681	11735	11698	11560
1.55	10418	11623	11650	11676	11852	11516
1.58	10200	11438	11407	11548	11397	11273
1.62	9996	11209	10982	11374	11372	11085
1.66	9797	10987	10863	11113	11002	10961
1.7	9404	10571	10463	10740	10817	10572
1.73	9074	9938	10211	10575	10403	10424
1.77	8764	9700	9875	10177	10022	10004
1.81	8528	9413	9425	9863	9561	9755
1.85	8222	9021	9183	9525	9234	9715
1.88	8162	8689	8886	9113	9010	9412
1.92	7957	8668	8916	9104	9043	9233
1.96	7811	8627	8748	8784	8801	9226
2	7939	8676	8707	8926	8896	9096
2.04	8141	8717	8746	8952	8878	9257
2.07	8140	8677	9076	8999	8994	9379
2.11	8360	9192	9332	9322	9192	9456
2.15	8427	9492	9567	9459	9593	9606
2.19	8858	9803	9866	10074	9851	10101
2.22	9398	10140	9935	10179	9929	10401
2.26	9484	10521	10679	10553	10423	10679
2.3	9803	10707	10734	10978	10674	11216
2.34	9896	10837	10953	11249	11188	11588
2.37	10047	11207	11417	11524	11375	11806
2.41	10292	11332	11467	11484	11799	11876
2.45	10166	11286	11692	11895	11971	12355
2.49	10379	11455	11480	11866	12015	12425
2.53	10337	11277	11492	12063	12316	12439
2.56	10221	11069	11338	12046	12049	12471
2.6	10115	11094	11267	11966	12329	12427
2.64	10042	10920	11095	12016	12140	12387
2.68	9938	10674	10912	11846	12111	12309
2.71	9923	10487	10766	11829	12077	12256
2.75	9663	10457	10524	11442	12011	12299
2.79	9432	10219	10460	11378	11737	12106
2.83	9283	10280	10291	11318	11510	12170
2.86	9206	9855	10031	11424	11629	12106
2.9	9058	9836	10048	11345	11379	11835
2.94	9113	9922	10123	11678	11305	11910
2.98	9020	10014	10099	11192	11265	11965
3.02	9070	9922	10183	11361	10897	12000
3.05	9278	9965	10354	11559	10904	11921
3.09	9351	10198	10590	11304	10943	11908
3.13	9524	10335	10548	11449	11020	11873

3.17	9702	10379	10818	11333	10976	11794
3.2	9862	10637	11063	11426	11211	11900
3.24	10184	11050	11131	11590	11038	11875
3.28	10367	11042	11388	11567	10924	11851
3.32	10178	11181	11349	11700	11050	11723
3.36	10530	11405	11623	11645	10869	11753
3.39	10513	11302	11659	11412	11041	11883
3.43	10639	11443	11465	11619	10798	11764
3.47	10649	11410	11653	11547	10885	11624
3.51	10619	11259	11697	11325	10860	11809
3.54	10382	11392	11577	11503	10672	11562
3.58	10307	11119	11519	11353	10591	11455
3.62	10370	10965	11227	11247	10505	11222
3.66	10263	10873	11163	11127	10505	11101
3.69	10142	10876	11094	11009	10574	11077
3.73	10022	10561	10904	10882	10233	10806
3.77	9854	10649	10801	10791	10180	11041
3.81	9801	10561	10742	10698	9840	10722
3.85	9633	10202	10685	10560	9830	10719
3.88	9795	10410	10630	10631	9882	10639
3.92	9811	10327	10553	10203	9833	10476
3.96	9906	10235	10508	10000	9629	10343
4	10030	10444	10758	10225	9447	10091
4.03	10163	10432	10742	9894	9538	10068
4.07	9982	10617	11009	10024	9471	10073
4.11	10235	10750	11005	9941	9685	9941
4.15	10417	10814	11297	9665	9737	9970
4.18	10518	10859	11207	9752	9715	9753
4.22	10538	10858	11304	9532	9716	9616
4.26	10599	11036	11516	9588	9707	9687
4.3	10572	11145	11491	9568	9529	9605
4.34	10616	11049	11511	9536	9527	9384
4.37	10862	11236	11525	9528	9394	9392
4.41	10631	11302	11588	9779	9481	9381
4.45	10748	11479	11644	9596	9439	9189
4.49	10707	11364	11604	9416	9341	9190
4.52	10522	11328	11561	9253	9367	9204
4.56	10672	11177	11432	9307	9357	8961
4.6	10443	11307	11522	9345	9308	8893
4.64	10451	11287	11534	9049	9290	8714
4.67	10566	11084	11537	9025	9183	8659
4.71	10652	11135	11468	8804	9118	8464
4.75	10508	11086	11614	8873	9155	8579
4.79	10522	11005	11581	8679	8922	8394
4.83	10580	11046	11521	8736	8804	8506

4.86	10499	10892	11447	8505	8816	8289
4.9	10802	10838	11509	8264	8850	8125
4.94	10612	11059	11439	8257	8763	8131
4.98	10676	10940	11369	8039	8609	8534
5.01	10950	11153	11625	7961	8388	8349
5.05	10857	11120	11760	7973	8534	8288
5.09	10957	11488	11832	7854	8368	8153
5.13	11076	11394	11819	7702	8289	8286
5.16	11188	11479	11937	7734	8249	8273
5.2	11253	11555	11899	7527	8270	8199
5.24	11314	11662	12375	7710	8106	7984
5.28	11254	11602	12279	7693	8033	8182
5.32	11512	11612	12368	7498	8081	8054
5.35	11598	12229	12332	7477	7973	7964
5.39	11430	12117	12470	7561	8147	7995
5.43	11334	12216	12571	7463	7973	7811
5.47	11495	12163	12581	7589	8014	7802
5.5	11542	12027	12628	7431	7993	7777
5.54	11746	12206	12712	7358	7929	7815
5.58	11723	12416	12776	7238	7759	7822
5.62	11698	12527	12574	7328	7872	7690
5.65	11788	12168	12747	7361	7856	7817
5.69	11874	12153	12704	7255	7605	7684
5.73	12043	12296	12938	7266	7770	7586
5.77	11960	12184	12728	7302	7722	7347
5.81	12189	12213	12754	7165	7634	7398
5.84	12095	12524	12671	7128	7601	7547
5.88	12131	12259	12969	7136	7550	7487
5.92	12078	12317	13025	7202	7634	7549
5.96	12059	12696	12983	7071	7515	7243
5.99	12178	12710	13388	7009	7444	7387

Fig 1A-B-C					
PSMD4	PSMD4	PSMD4	PSMD12	PSMD12	PSMD12
6427	6698	7013	8214	7806	7961
7319	7609	7657	8870	8759	8873
8404	9030	9039	9776	9469	10076
10048	10256	10229	10969	10778	11223
11382	11942	11466	12342	12257	12082
12716	13332	12816	14026	13620	12925
14053	14486	14104	15091	14289	13844
15210	15638	15138	16200	15319	14327
15986	16414	15883	17244	16309	14830
17079	17262	16780	18116	17160	15102
17528	17651	17181	18639	17749	15232
18027	17933	17627	19068	18224	15467
17392	18124	18018	18869	17949	15167
17280	17448	17688	18669	18053	14833
16640	16839	16742	18245	17521	14413
15576	16120	15843	17082	16720	13732
14299	14637	14938	16065	15831	12756
13041	13163	13428	14660	14400	11916
11614	11873	12454	13345	13030	10845
10085	10271	10765	11824	11735	9647
8599	8846	9431	10375	10589	8589
7451	7414	8191	8991	9213	7447
6369	6537	7019	7896	8075	6764
5489	5635	6096	6963	7127	5937
4662	4798	5137	6130	6403	5507
4267	4154	4646	5592	5961	4919
3891	3811	4265	5121	5614	4729
3686	3670	4033	5098	5483	4489
3662	3781	4042	5140	5498	4552
3812	3919	4232	5343	5662	4783
4238	4093	4430	5642	6111	5122
4487	4555	5096	6308	6561	5387
5266	5139	5611	6784	7148	5873
5670	5573	6122	7308	7696	6447
6215	6251	6700	8010	8244	6965
6974	6945	7366	8445	8705	7350
7347	7475	7893	8799	9013	7618
7770	8063	8274	8733	9406	8142
8281	8247	8601	9078	9487	8111

8574	8384	8848	8961	9718	8071
8585	8393	8830	8879	9720	8291
8566	8482	8754	8600	9439	8253
8244	8309	8690	8280	9185	8051
8053	8174	8518	8068	9022	7920
7877	7760	8115	7770	8668	7631
7227	7425	7787	7189	8360	7408
7022	6967	7322	6920	8076	7190
6667	6423	7026	6499	7664	7028
6071	5916	6595	5954	7318	6736
5734	5582	6188	5793	7190	6690
5341	5319	5698	5502	6872	6471
4863	5097	5611	5269	6611	6420
4770	4721	5292	5339	6596	6521
4553	4513	5172	5240	6467	6345
4510	4521	5143	5400	6726	6547
4556	4613	5199	5543	6880	6588
4734	4919	5313	5954	7031	6939
4969	5195	5705	6206	7246	7214
5412	5464	5939	6444	7562	7581
5665	5856	6147	6857	7887	7796
5976	6255	6630	7218	8220	8108
6453	6726	7150	7537	8527	8211
6873	6967	7398	7850	8625	8635
7444	7581	7753	8027	8861	8607
7413	7860	8070	7987	9024	8771
7815	8346	8397	8063	9513	8998
8066	8400	8759	8062	9678	9027
8083	8630	8777	8100	9419	8990
8100	8550	8764	7844	9546	8946
8043	8509	8719	7653	9118	8665
7817	8485	8667	7502	8899	8685
8002	8233	8622	7178	8707	8591
7673	7784	8334	6912	8507	8459
7452	7668	8541	6605	8159	8116
6911	7426	8673	6369	7988	7951
6802	7066	8207	6380	7768	7837
6637	6711	7896	5989	7562	7749
6254	6390	7673	5947	7364	7595
5973	6236	7404	5812	7382	7725
5846	5934	7045	5736	7105	7738
5760	5854	6831	5763	7149	7612
5683	5679	6769	5856	7194	7646
5660	5646	6737	5850	7214	7621
5646	5636	6837	6084	7348	7811

5649	5725	6857	6175	7363	7897
5970	5973	6766	6384	7497	7749
6049	6068	7033	6512	7524	8019
6162	6267	7179	6506	7774	8011
6539	6539	7461	6508	7844	8178
6766	6733	7312	6773	8038	8254
6969	7024	7739	6697	8040	8254
7142	7115	7880	6737	8020	8215
7360	7309	8122	6830	8072	8307
7740	7578	8117	6771	8086	8319
7326	7603	8473	6577	8077	8219
7549	7681	8402	6401	7930	8206
7530	7738	8392	6302	7974	8243
7461	7823	8378	6273	7797	8119
7340	7723	8517	6132	7638	8020
7463	7669	8326	5951	7514	7808
7196	7423	8151	5872	7302	7908
7070	7306	8044	5855	7445	7732
6908	7083	7981	5943	7339	7628
6788	6828	7793	5721	7076	7489
6710	6651	7599	5648	6946	7451
6506	6483	7564	5511	6867	7424
6497	6367	7471	5622	6779	7431
6268	6310	7257	5538	6793	7270
6244	6239	7095	5683	6935	7339
6205	6154	7080	5689	6908	7412
6184	6129	6906	5733	6813	7401
6750	6183	7695	5738	6935	7316
6822	6167	7596	5810	6775	7475
6830	6392	7743	5884	6926	7368
6983	6504	7787	6155	6978	7530
6961	6553	8529	6225	7026	7357
7195	6702	8746	6239	6935	7467
7292	6737	8738	6353	7110	7540
7230	6904	8855	6168	7042	7557
7633	7067	9038	6258	7057	7578
7865	6999	9117	6113	7242	7411
8028	7099	9129	6188	6963	7409
8049	8491	9306	6122	7097	7467
7982	8545	9292	5951	7074	7262
8012	8629	9462	5813	6912	7207
7932	8628	9420	5764	6879	7309
8005	8481	9448	5795	6951	7154
7963	8573	9245	5742	6859	7141
7917	8556	9333	5747	6848	7276

7945	8411	9356	5646	6863	7184
7899	8374	9226	5607	6736	7098
7753	8357	9170	5716	6566	7091
7504	8318	9165	5533	6483	7083
7601	8051	8921	5704	6597	7099
7474	8155	8891	5675	6656	7204
7482	7908	8906	5794	6590	6998
7312	7800	8719	5675	6765	7038
7282	7823	8837	5887	6594	7004
7401	7874	8817	5880	6602	7108
7218	7925	8766	5836	6692	6922
7274	7895	8828	6108	6755	7164
7285	7860	8974	5963	6813	7029
7336	7917	8841	6122	6837	7185
7455	7943	8950	6094	6699	7184
7429	7966	8940	6128	6707	7102
7583	8077	9079	5917	6713	7207
7622	7906	9082	6084	6859	7153
7775	8188	9028	6096	6857	6964
7560	8143	9011	6198	6849	7064
7626	8171	8934	6057	7266	7133
7767	8252	8865	6032	7164	7041
7712	8229	9040	6147	7181	6969
7838	8339	9168	5980	7214	7047
7783	8404	9060	6027	7061	6970
7816	8413	8971	5945	7230	7100
7789	8464	8881	5963	7242	6875
7886	8288	8931	5987	7105	6942
7735	8318	8829	5930	7157	6821
7812	8268	8901	5858	7185	7052
7587	8435	8951	6051	7139	6996

**Fig1D**	siRNA	Baseline	Amplitude	Period
U2 OS *Per2:dLuc*	Control	10801 ± 4066	2403 ± 410	24.9 ± 0.2 h
	PSMD11 KD	5391 ± 1275	2515 ± 312	NA
	PSMD4 KD	7111 ± 1755	1929 ± 603	24.3 ± 0.3 h
	PSMD12 KD	4075 ± 1522	3347 ± 1568	25.5 ± 0.3 h

Fig1E

siNEG	*siPSMD11*	*siNEG*	*siPSMD4*	*siNEG*	*siPSMD12*
1	0.2	1	0.035	1	0.085
0.95	0.08	0.85	0.029	0.98	0.21
0.9	0.085	0.9	0.1	0.9	0.225

Fig2A	siNEG			siPSMD11		
PER1	1.06		0.92	1.22703136		2.00446791
PER2	1.06	0.92		2.4344447	2.00446791	
CRY1	1	1.06	0.92	0.84875502	1.06678561	0.96358174
CRY2	1.05	1.06	0.92	2.55972589	2.14316539	1.57310084

Fig2B	
PER2	CRY2
1.00228934	1.00166847
1.4093663	0.86265139
1.87308946	1.196848
1.67741754	1.06813462
2.4968308	1.49728482
2.9179426	1.76030583
3.69728598	2.54133568
2.77530927	2.8547124

**FIG3A**	PER2		
30	0.891306	1.01512424	1.09363948
36	0.89070049	0.5466605	0.96703017
42	1.09995489	0.27964688	1.26922888
48	1.92844133	1.32492195	1.48214111
54	0.891306	1.01512424	1.09363948

**FIG3A**	CRY1		
30	1.07956725	0.29627127	1.62431424
36	0.95877519	0.27461439	1.31354998
42	0.31849737	0.42801241	1.52412923
48	1.45841578	2.76561509	2.74698908
54	1.07956725	0.29627127	1.62431424

**FIG3A**	PSMD11		
30	0.99630626	1.10743608	0.89641074
36	1.27338598	1.53667649	0.79713791
42	1.24699807	1.39910491	1.02042863
48	0.67433144	1.1655748	0.69321507
54	0.99630626	1.10743608	0.89641074

FIG3C

CYTOPLASMIC FRACTION						
30	1.00000984	0.99889719	1.00006265	1.01261238	0.98405646	1.0942154
36	0.24768574	0.68787643	1.36618011	1.654162	0.801379	2.6345007
42	1.0344125	0.90623872	1.32889713	1.65918613	0.8461203	2.79402
48	0.64788389	0.78961804	1.0524559	0.86001775	0.52377048	1.1087953

CYTOPLASMIC FRACTION							
30	0.9347385	1.01694915	1.00004622	0.99436	0.99985167	1.00520558	1.06337529
36	1.6461087	2.00968068	1.76976303	2.08889495	1.58534606	1.19190274	1.21524986
42	1.4469459	0.87948356	1.80991713	1.37028161	1.60610682	0.72238708	1.36228329
48	1.2335648	1.13088068	0.83232985	1.1494029	1.23658106	0.94159504	1.16923243

CYTOPLASMIC FRACTION			
30	0.986907	1.038522	0.963894
36	2.933155	1.680011	0.99903
42	1.467047	2.126353	1.034515
48	1.699871	2.638496	1.384588

FIG3C

NUCLEAR FRACTION						
30	5.77726216		0.98476957		0.32642148	
36	5.32202259	1.23115497	0.29117723	0.50092749	0.3095945	0.43693095
42	4.34032659	1.50714985	0.30747013	0.48185563	0.2807897	0.39740135
48	3.60214106	1.59634544	0.35624753	0.25984811	0.23434063	0.4288802

NUCLEAR FRACTION							
30	4.095107		6.38875037		7.16603879		4.944958
36	2.0806116	0.68027441	4.5570524	3.23405978	5.28725864	2.07956442	4.77077743
42	1.8241187	1.05136305	3.51993862	2.24991398	6.15573712	1.9441492	3.18347143
48	1.1583083	1.34683136	2.88458419	2.12445387	3.65486167	2.38755938	2.18756943

NUCLEAR FRACTION			
30		12.5153837	
36	4.64185756	7.98270368	3.53040545
42	3.41890822	8.17552684	3.95286636
48	5.08916844	1.51376526	2.59969424

**Fig4B-decreased**	siNEG			siPSMD11			
IQGAP1	1	1.10006375	0.89291	1.00881606	0.81223325	1.100069	cytosolic
KPNB1	0.94399783	0.93067932	1.12542914	2.84770607	0.59757972	0.65440998	
CRY1	1.07708979	0.96571054	0.95730953	2.36790762	1.05772442	1.27707309	
GSK3β	0.73892478	1.25456111	1.00738751	2.82078173	2.41654399	0.98521154	
CSNK1ε	0.86334045	0.73101158	1.40569712	1.32700463	1.07149794	1.23646732	
IQGAP1	1.9333115	2.39351125	2.00160306	0.74286175	1.80281675	1.26859983	nuclear
KPNB1	2.44123174	2.55219143	2.25373463	1.12613015	1.51274584	1.00418402	
CRY1	3	1.73126547	2.5	1.40155373	0.74712223	1.48971565	
GSK3β	7.44476494	6.97860578	7.03316756	2.47653324	2.73616118	2.24757119	
CSNK1ε	3.82766894	3.82351184	3.38171333	1.38688163	2.1193209	1.49107488	

**Fig4B-not changed**	siNEG			siPSMD11			
PER2	1.07388301	1.01547806	0.91167647	1.24465011	2.05012512	0.60705182	cytosolic
CSNK1δ	0.99808044	1.09260725	0.90942254	0.89923635	2.7895385	2.28972046	
CaN	1.06220491	0.75466316	1.18337509	3.86124175	1.29739544	1.35131754	
PER2	4.161805	3.61781886	2.74255836	3.41957816	2.74776424	4.44743651	nuclear
CSNK1δ	9.09932324	6.72819911	8.62760179	8.31091708	7.60765988	5.81429494	
CaN	4.38287877	3.50877193	3.41668772	1.34675246	2.49321123	2.22467263	

**Fig4B-increased**	siNEG			siPSMD11			
CRY2	1.0039862	0.9337489	1.06246367	2.70436487	2.1951536	1.51912712	cytosolic
CRY2	0.95369557	0.93690137	1.10575116	1.39706463	1.88130456	1.70108847	nuclear
